# The Pheromone Module SteC-MkkB-MpkB-SteD-HamE Regulates Development, Stress Responses and Secondary Metabolism in *Aspergillus fumigatus*

**DOI:** 10.3389/fmicb.2020.00811

**Published:** 2020-05-07

**Authors:** Dean Frawley, Maria C. Stroe, Berl R. Oakley, Thorsten Heinekamp, Maria Straßburger, Alastair B. Fleming, Axel A. Brakhage, Özgür Bayram

**Affiliations:** ^1^Department of Biology, Fungal Genetics and Secondary Metabolism Laboratory, Maynooth University, Maynooth, Ireland; ^2^Department of Molecular and Applied Microbiology, Hans Knöll Institute (HKI), Leibniz Institute for Natural Product Research and Infection Biology, Jena, Germany; ^3^Department of Molecular Biosciences, University of Kansas, Lawrence, KS, United States; ^4^Transfer Group Antiinfectives, Leibniz Institute for Natural Product Research and Infection Biology, Hans Knöll Institute (HKI), Jena, Germany; ^5^Department of Microbiology, School of Genetics and Microbiology, Moyne Institute of Preventive Medicine, Trinity College Dublin, Dublin, Ireland; ^6^Department of Microbiology and Molecular Biology, Institute of Microbiology, Friedrich Schiller University, Jena, Germany

**Keywords:** *Aspergillus fumigatus*, gliotoxin, pheromone module, secondary metabolism, MAP Kinases, stress responses, asexual sporulation

## Abstract

In order for eukaryotes to efficiently detect and respond to environmental stimuli, a myriad of protein signaling pathways are utilized. An example of highly conserved signaling pathways in eukaryotes are the mitogen-activated protein kinase (MAPK) pathways. In fungi, MAPK pathways have been shown to regulate a diverse array of biological processes, such as asexual and sexual development, stress responses and the production of secondary metabolites (SMs). In the model fungus *Aspergillus nidulans*, a MAPK pathway known as the pheromone module is utilized to regulate both development and SM production. This signaling cascade consists of the three kinases SteC, MkkB, and MpkB, as well as the SteD adaptor protein and the HamE scaffold. In this study, homologs of each of these proteins have been identified in the opportunistic human pathogen *A. fumigatus.* By performing epitope tagging and mass spectrometry experiments, we have shown that these proteins form a pentameric complex, similar to what is observed in *A. nidulans.* This complex has been shown to assemble in the cytoplasm and MpkB enters the nucleus, where it would presumably interact with various transcription factors. Pheromone module mutant strains exhibit drastic reductions in asexual sporulation, vegetative growth rate and production of SMs, such as gliotoxin. Mutants also display increased sensitivity to cell wall and oxidative stress agents. Overall, these data provide evidence of the existence of a conserved MAP kinase signaling pathway in *Aspergillus* species and suggest that this pathway is critical for the regulation of fungal development and secondary metabolism.

## Introduction

*Aspergillus fumigatus* is a saprophytic fungus that is ubiquitous in the environment and is an opportunistic human pathogen (Latge, 1999). This species reproduces predominately via the production of hydrophobic conidia that can easily spread throughout the air, allowing for the rapid colonization of new environments (Dagenais and Keller, 2009). The conidia of this fungus can pose severe threats to human health, as these spores are commonly inhaled daily and can germinate in the alveoli in the lungs (Latge, 1999). Within 4–6 h of colonization, conidia can spread throughout the lungs, resulting in the development of invasive pulmonary aspergillosis (van de Veerdonk et al., 2017). Immunocompromised individuals, such as patients that are undergoing chemotherapy or organ transplantations have a much higher risk of developing pulmonary aspergillosis and the mortality rate in these individuals is generally over 50%, reaching as high as 95% in specific situations (Latge, 1999, 2001; Maschmeyer et al., 2007; Balloy and Chignard, 2009; McCormick et al., 2010).

A myriad of virulence factors contribute to the survival and spread of *A. fumigatus* spores in the human body, making *A. fumigatus* a highly adaptable pathogen. For example, *A. fumigatus* utilizes various systems that aid in the detoxification of reactive oxygen species that are produced by phagocytic immune cells like neutrophils and macrophages (Abad et al., 2010; Hillmann et al., 2016). Another virulence factor is the fungal cell wall, which is the main defense and source of structural integrity for *A. fumigatus* cells as they colonize the lungs (Abad et al., 2010). The cell wall retains high plasticity and its composition is readily altered to adapt to various environmental conditions and cell stressors, allowing for *A. fumigatus* spores to avoid the body’s natural defense mechanisms (van de Veerdonk et al., 2017). *A. fumigatus* growth and virulence is greatly influenced by the ability of this species to produce various bioactive compounds known as secondary metabolites (SMs), which can possess a myriad of properties. *A. fumigatus* has 40 predicted SM core synthase enzyme-encoding genes, 19 of which have been shown to produce downstream products (Romsdahl and Wang, 2019). The production of gliotoxin, a SM with immunosuppressive properties is a major contributor to virulence (Hof and Kupfahl, 2009) and is implicated in 96% of cases of *A. fumigatus* infections (Ghazaei, 2017). Gliotoxin inhibits the activity of various enzymes including nicotinamide adenine dinucleotide phosphate (NADPH) oxidases and alcohol dehydrogenases. Gliotoxin is also capable of inducing apoptosis and inhibiting various functions of macrophages and neutrophils (Gardiner et al., 2005; Spikes et al., 2008). As a result, gliotoxin production enables fungal growth and colonization of host tissue *via* suppression of the immune system (Ghazaei, 2017).

In order for fungal species like *A. fumigatus* to regulate their development, stress responses and secondary metabolism in response to external stimuli, a variety of protein signaling pathways are utilized (Bayram et al., 2008, 2012; Elramli et al., 2019). Mitogen-activated protein kinase (MAPK) pathways are highly conserved signaling cascades in eukaryotes that are critical for the regulation of various biological processes such as cell growth and immune responses, to name a few (Marshall, 1994; Schaeffer and Weber, 1999; Widmann et al., 1999; Shaul and Seger, 2007; Rincon and Davis, 2009). In a general MAPK pathway, stimulus detection at a receptor leads to the activation of three protein kinases, often termed MAPKKK/MAP3K, MAPKK/MAP2K, and MAPK, which phosphorylate each other sequentially. The MAPK translocates into the nucleus when phosphorylated and activates various transcription factors and regulators, which in turn, modulate numerous biological processes (Marshall, 1994; Widmann et al., 1999; Saito, 2010).

In the model ascomycete fungus *A. nidulans*, a MAPK pathway known as the pheromone module has been characterized (Bayram et al., 2012; Frawley et al., 2018). This pathway consists of three kinases, known as SteC (MAP3K), MkkB (MAP2K), and MpkB (MAPK), as well as the SteD adaptor protein and the HamE scaffold protein. These proteins form a pentameric complex that assembles at the plasma membrane and hyphal tips in response to pheromone signaling. Once assembled, kinase phosphorylation enables transduction of a signal downstream, via translocation of MpkB into the nucleus where it interacts with various transcription factors such as SteA and the velvet protein VeA to modulate biological processes. Activation of the SteA transcription factor results in the positive regulation of hyphal fusion and formation of cleistothecia, which are sexual reproductive structures. VeA activation results in assembly of the trimeric velvet complex (VeA-VelB-LaeA) which regulates secondary metabolism (Vallim et al., 2000; Atoui et al., 2008; Bayram et al., 2008, 2012; Sarikaya Bayram et al., 2010). Characterization of this pheromone module signaling pathway led to the identification of a homologous signaling module in the saprophytic fungus *A. flavus* (Frawley et al., 2020). This species is a prolific producer of the carcinogen aflatoxin B1 and causes contamination of a wide array of agricultural crops (Lewis et al., 2005; Yu et al., 2005; Amare and Keller, 2014; Rushing and Selim, 2019). The *A. flavus* pheromone module consists of SteC, MkkB, MpkB, and SteD. However, HamE was not shown to interact with the proteins of this pathway. This tetrameric complex was shown to assemble in the cytoplasm and is essential for the regulation of asexual sporulation, sclerotia formation and aflatoxin B1 production (Frawley et al., 2020).

Both secondary metabolism and various methods of fungal development are co-regulated *via* pheromone module signaling in *A. nidulans* (Bayram et al., 2012; Frawley et al., 2018) and *A. flavus* (Frawley et al., 2020). This information, coupled with the recent identification of the MpkB homolog in *A. fumigatus* (Manfiolli et al., 2019), led to the proposal that *A. fumigatus* may also utilize a similar mechanism of regulation to control its developmental programs and SM production. In this work, homologs of the remaining pheromone module proteins (SteC, MkkB, SteD, and HamE) have been identified in *A. fumigatus.* Using a genetic and proteomic approach, we detected physical interactions between these proteins. In combination with confocal imaging, these data suggest that these proteins form a MAP kinase pheromone module in the cytoplasm and that MpkB enters the nucleus, similar to what is observed in both *A. nidulans* (Bayram et al., 2012; Frawley et al., 2018) and *A. flavus* (Frawley et al., 2020). This work also provides evidence that the pheromone module is critical for the regulation of asexual sporulation, cell stress responses and secondary metabolism. Overall, the data from this study suggests that the pheromone module is a highly conserved signaling pathway that is critical for the regulation of development and secondary metabolism in *Aspergillus* species.

## Materials and Methods

### Strains, Growth Media and Culturing Conditions

Fungal strains used in this study are listed in Supplementary Table S1. The *Aspergillus fumigatus* CEA17 (*pyrG*+) and CEA17 (*pyrG*Δ) strains served as wild-type hosts for all deletions and epitope taggings. Various plasmids used for the knock-out and epitope tagging experiments are listed in Supplementary Table S2. Plasmids were cloned into Stellar (Clontech) competent *Escherichia coli* cells and these cells were cultured in LB medium (supplemented with 100 μg/ml ampicillin) and SOC media. To induce asexual sporulation of fungal strains, Glucose Minimal Medium (GMM) agar plates were used. To promote vegetative growth, liquid complete medium, Czapek-Dox medium and Sabouraud medium were used. Details of the ingredients of each medium are provided in the Supplementary Information.

### Phenotypic Assays

Strains were point inoculated (5 × 10^3^ spores) in triplicate on GMM agar plates containing appropriate supplements. Plates were incubated in the presence of light for 4 days to induce asexual sporulation. All incubations were performed at 37°C. Stereomicroscopic images were captured using an Olympus szx16 microscope with Olympus sc30 camera. Digital pictures were taken and processed with the Cell Sens Standard software (Olympus). Quantifications of colony diameter and asexual conidiation were performed using three independent biological replicates. Bar charts represent the mean values ± s.d. *P*-values were calculated by performing unpaired Student’s *t*-tests (^∗^*P* < 0.05; ^∗∗^*P* < 0.01; ^∗∗∗^*P* < 0.001), using Graphpad Prism Version 6.

For testing the stress responses of *A. fumigatus* strains, strains were inoculated on GMM agar plates containing appropriate supplements and the following stress agents were used: Congo Red (20, 30, 50 μg/ml), H_2_O_2_ (2, 3, 4 mM) and NaCl (0.5, 1 and 1.5 M). All plates were incubated at 37°C for 3 days.

### GFP/HA-Trap and Sample Preparation for LC-MS Protein Identification

Isolation and preparation of GFP and HA fusion proteins for mass spectrometry analysis was performed as explained in detail (Bayram et al., 2008). Detailed descriptions of methods used are given in the provided Supplementary Information.

### RP-HPLC Analysis of Gliotoxin Levels

Detailed information on culturing conditions and preparation of samples for RP-HPLC analysis is provided in Supplementary Information. 3 biological replicates were prepared per strain and data is presented as a bar chart, with the bars representing the mean ± s.d. *P*-values were calculated by performing unpaired Student’s *t*-tests (^∗^*P* < 0.05; ^∗∗^*P* < 0.01), using the Graphpad Prism Version 6.

### Extraction of Fungal Compounds and LC-MS Analysis

Strains were inoculated in triplicate in 40 ml of liquid GMM at a concentration of 5 million spores/ml and incubated for 48 h on a shaker at 37°C. The culture broth containing fungal mycelium was homogenized using an ULTRA-TURRAX (IKA-Werke, Staufen, Germany). Homogenized cultures were extracted twice with a total of 100 ml ethyl acetate, dried with sodium sulfate and concentrated under reduced pressure. For LC-MS analysis, the dried extracts were dissolved in 1 ml of methanol and loaded onto an ultrahigh-performance liquid chromatography (LC)–MS system consisting of an UltiMate 3000 binary rapid-separation liquid chromatograph with photodiode array detector (Thermo Fisher Scientific, Dreieich, Germany) and an LTQ XL linear ion trap mass spectrometer (Thermo Fisher Scientific, Dreieich, Germany) equipped with an electrospray ion source. The extracts (injection volume, 10 μl) were analyzed on a 150 mm by 4.6-mm Accucore reversed-phase (RP)-MS column with a particle size of 2.6 μm (Thermo Fisher Scientific, Dreieich, Germany) at a flow rate of 1 ml/min, with the following gradient over 21 min: initial 0.1% (v/v) HCOOH-MeCN/0.1% (v/v) HCOOH-H_2_O 0/100, which was increased to 80/20 in 15 min and then to 100/0 in 2 min, held at 100/0 for 2 min, and reversed to 0/100 in 2 min.

### Confocal Microscopy

Conidia were cultured in eight-chambered cover glasses (Lab-Tek; Thermo Fisher Scientific) and incubated at 30°C for various durations in 400 μL of liquid GMM, containing appropriate supplements. Additional details for DAPI staining, immunostaining and confocal imaging are provided in the Supplementary Information.

## Results

### The SteC, MkkB, MpkB, SteD, and HamE Homologs Physically Interact in *A. fumigatus*

**FIGURE 1:**
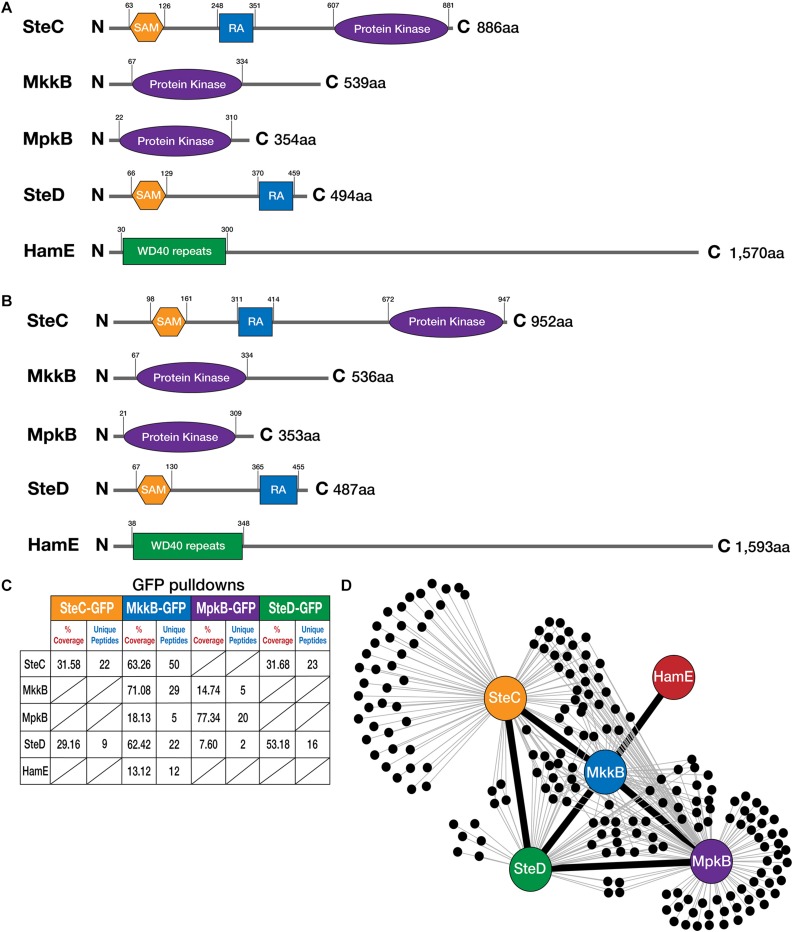
Detection of interactions between pheromone module proteins in *A. fumigatus.* **(A)** Schematic diagrams illustrating the structures of the pheromone module proteins in *A. nidulans.* Amino acids (aa). N (N-terminal), C (C-terminal). SAM (Sterile Alpha Motif), RA (Ras-associated). Detection of protein sizes and domains were performed using a combination of ScanProsite (de Castro et al., 2006) and InterPro software (Mitchell et al., 2019). **(B)** Schematics illustrating the structures of the pheromone module proteins in *A. fumigatus.* Reciprocal BLAST searches were performed to detect protein homologs (Altschul et al., 1990). **(C)** GFP-pulldowns and LC-MS/MS analysis of the pheromone module kinases and SteD. GFP-tagged proteins are given at the top of the table and co-purified proteins are given on the left-hand side. The percentage of coverage and unique peptides of each detected protein are displayed. Two biological replicates of each strain were used. Strains were cultured vegetatively for 24 h in complete media. **(D)** Interaction network of the pheromone module components based on unique peptides detected in each GFP pulldown. Each black dot represents a protein detected in two independent biological replicates but not in the wild type.

To determine whether *A. fumigatus* possesses homologs of the pheromone module proteins that are present in *A. nidulans*, reciprocal BLAST searches (Altschul et al., 1990) were performed and the ASPGD website was utilized^1^. Homologs of all five members of the *A. nidulans* pheromone module were found to exist in the *A. fumigatus* genome. According to BLAST and ASPGD, the *A. fumigatus* SteC homolog (Afu5g06420) exhibits 78.04% sequence similarity to the *A. nidulans* protein, while *A. fumigatus* MkkB (Afu3g05900), MpkB (Afu6g12820) (Manfiolli et al., 2019), SteD (Afu2g17130) and HamE (Afu5g13970) exhibit 80.19, 98.59, 75.65, and 64.57% sequence similarity, respectively. To determine the sizes of these *A. fumigatus* proteins and the domains they possess in comparison to the *A. nidulans* proteins, “ScanProsite” (de Castro et al., 2006) and “InterPro” (Mitchell et al., 2019) software was used (Figures 1A,B).

It was found that SteC in both *A. nidulans* and *A. fumigatus* possesses a SAM domain at the N-terminus. In *A. nidulans*, this SAM domain is located between amino acid residues 63 and 126, while for *A. fumigatus*, it is present between amino acids 98 and 161. SteC in both species also contains a RA domain and a protein kinase domain. The RA domain in *A. nidulans* is located between amino acids 248 and 351, while the RA domain in *A. fumigatus* is located at amino acids 311 and 414. The protein kinase domain is located at amino acids 607 and 881 in *A. nidulans*, while the protein kinase domain in *A. fumigatus* is located at amino acids 672 and 947. According to ASPGD, the sequence provided for *A. fumigatus* SteC is 1,007 amino acids in length, considerably larger than the *A. nidulans* sequence (886 amino acids). However, attempts at tagging this sequence at the C-terminus with various epitope tags proved to be unsuccessful. This suggested that the sequence provided on ASPGD is incorrect. Pair-wise sequence alignment of the *A. nidulans* and *A. fumigatus* protein sequences using the Smith-Waterman algorithm (Madeira et al., 2019) led to the determination of the extent of homology (Supplementary Figure S3). This alignment revealed the presence of an alternate stop codon, ahead of the stop codon predicted on ASPGD. Tagging of the SteC sequence from this stop codon proved to be successful (Supplementary Figure S1B). This leads us to conclude that the *A. fumigatus* protein sequence is 952 amino acids in length, as opposed to 1,007 amino acids.

MkkB in both *A. nidulans* and *A. fumigatus* possesses a protein kinase domain. This domain extends from amino acids 67–334 in both of these species, signifying high conservation between these two orthologs. MpkB in both species also possesses a protein kinase domain at very similar residues. In *A. nidulans*, this domain is present at amino acids 22–310, while in *A. fumigatus*, this domain extends from amino acids 21–309. The SteD adaptor in both species contains SAM and RA domains. The SAM domains in *A. nidulans* and *A. fumigatus* are located at amino acids 66–129 and 67–130, respectively. The RA domains are located at amino acids 370–459 and 365–455 in *A. nidulans*, and *A. fumigatus*, respectively. Lastly, the HamE protein consists of WD40 repeats at the N-terminus of both proteins between amino acids 30–300 and 38–348 in *A. nidulans* and *A. fumigatus*, respectively.

To assess protein-protein interactions between these pheromone module proteins, the C-terminal ends of the *steC, mkkB, mpkB*, and *steD* genes were fused to an *sgfp* epitope tag (Supplementary Figure S1). All attempts to successfully detect the *hamE* gene tagged with *sgfp via* western blotting and mass spectrometry (MS) failed. We therefore coupled the C-terminus of the *hamE* gene to a *3xha* epitope tag (Supplementary Figure S2). Each tagged protein was immunoprecipitated from strains that had undergone vegetative growth for 24 h. These samples were run on a MS to detect the tagged proteins and their interaction partners (Figure 1C). It was found that SteC-GFP pulldowns co-purified the adaptor protein SteD (Supplementary Table S4), MkkB-GFP pulldowns co-purified SteC, MpkB, SteD, and HamE (Supplementary Table S5), MpkB-GFP pulldowns co-purified MkkB and SteD (Supplementary Table S6) and SteD-GFP pulldowns co-purified SteC (Supplementary Table S7). Despite HamE being detectable in purifications of MkkB-GFP, HamE-HA pulldowns did not co-purify any pheromone module components (Supplementary Table S8) and so this interaction may be transient or the binding affinity may be too low to allow co-purifications under the conditions we used. Taken together, this interactome data provides evidence that a complex of at least four proteins is assembled in *A. fumigatus* (Figure 1D). This complex consists of the three kinases SteC, MkkB, and MpkB, as well as the adaptor protein SteD and possibly the HamE scaffold protein.

### Each Pheromone Module Protein Is Critical for the Regulation of Asexual Sporulation and Vegetative Growth

In order to assess whether the pheromone module protein homologs in *A. fumigatus* contribute to the regulation of asexual sporulation, mutant strains were generated. The respective *steC, mkkB, mpkB, steD*, and *hamE* gene open reading frames were deleted (Supplementary Figures S1, S2) by replacing these genomic regions with either the pyrithiamine resistance gene (*ptrA*) or the *A. fumigatus pyrG* gene. To determine whether phenotypic differences observed in the mutant strains were due to the deletion of specific genes and not secondary abnormalities, complementation strains were made. A functional copy of each gene open reading frame, including the promoter and terminator regions were reinserted into the respective mutant strains to restore the wild-type phenotype.

**FIGURE 2:**
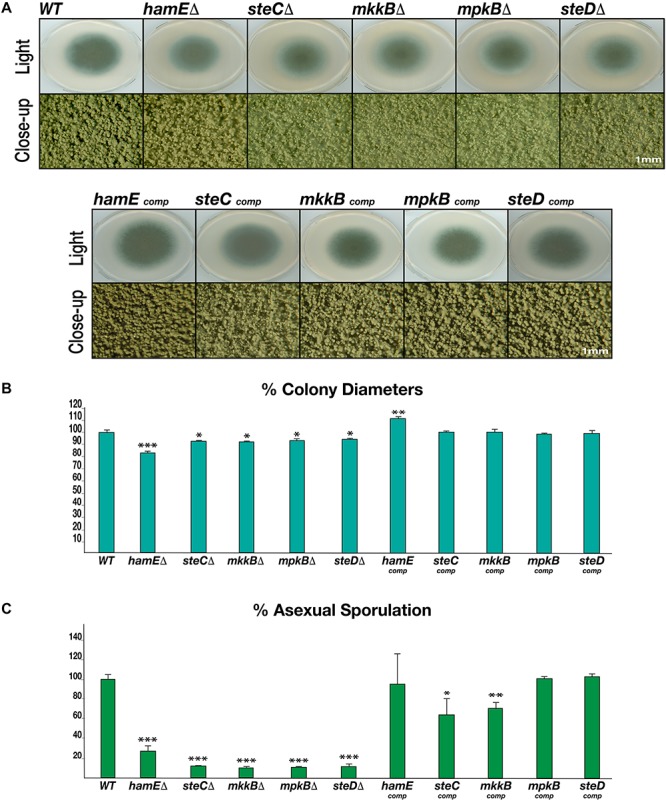
Rates of asexual sporulation and vegetative growth in deletion and complementation strains. **(A)** Asexual phenotypes of deletion and complementation strains. Each strain was spot inoculated (5 × 10^3^ spores) in triplicate on GMM agar plates containing appropriate supplements. Wild type refers to the CEA17 strain. All plates were incubated at 37°C for 4 days. The Olympus szx16 microscope with an Olympus sc30 camera was used to capture close-up images at 2 × magnification. **(B)** Rates of vegetative growth for each strain. The average values from three independent biological replicates were plotted ± s.d. as a percentage of the WT strain. *P-*values were calculated by performing unpaired Student’s *t*-tests (*P < 0.05; **P < 0.01; ***P < 0.001). **(C)** Rates of asexual sporulation for each strain calculated by performing spore counts of colonies on plates.

Each mutant and complementation strain were spot inoculated on GMM agar plates. These plates were incubated in the presence of light for 4 days to induce asexual reproduction and production of conidia (Figure 2A). For each of the five mutant strains, a dramatic reduction in sporulation was observed, similar to what is observed in both *A. nidulans* (Bayram et al., 2012; Frawley et al., 2018) and *A. flavus* (Frawley et al., 2020), with the exception of the *A. flavus hamE* mutant, which did not show any defects in asexual reproduction. For the *A. fumigatus* mutants, the average values of conidia produced were expressed as a percentage of the CEA17 wild-type average, which was chosen to represent 100% production (Figure 2C). The average percentage range for these mutants was between 10.16 and 27.02%. The complementation of each gene restored the ability of these strains to undergo asexual sporulation to a similar degree to that of the wild type. The average percentage range of sporulation for the complementation strains was between 63.06 and 102.89%.

To determine whether the pheromone module proteins contribute to regulating vegetative growth in *A. fumigatus*, the colony diameters of each strain were measured (Figure 2B). It was observed that each mutant exhibited a significantly smaller colony diameter in comparison to the CEA17 strain, with the *hamE* mutant displaying the highest degree of reduction. Aside from the *hamE* mutant phenotype, these data support the findings in *A. nidulans*, where the deletion of either *steC, mkkB, mpkB*, or *steD* results in a dramatic reduction in vegetative growth (Frawley et al., 2018). However, these results contradict the findings in *A. flavus* since no reductions in the rates of hyphal extension were observed in any of these mutants (Frawley et al., 2020). For the *A. fumigatus* deletion strains, the average percentage range of colony diameters was between 83.22–94.61%. Complementation of each gene restored the wild type phenotype and the average percentage range of hyphal extension was 98.2–111.37%.

Taken together, these data suggest that the pheromone module proteins are essential for the regulation of both asexual sporulation and vegetative hyphal growth. These findings also suggest that these five proteins may act as a complex to regulate these processes due to the similarities of the mutant phenotypes.

### The Pheromone Module Proteins Contribute to the Regulation of Cell Wall and Oxidative Stress Responses

Fungi like *A. fumigatus* utilize multiple MAPK pathways to respond to various cell stressors, such as cell wall, osmotic and oxidative stresses (Rispail et al., 2009; Hamel et al., 2012). To determine the relevance of the pheromone module proteins with respect to the responses to cell stressors, each mutant and complementation strain was spot inoculated on GMM agar plates containing various exogenous stress agents. The radial growth phenotypes of each strain were compared to the wild-type phenotypes. In order to assess whether the pheromone module proteins play a role in the response to osmotic stress, each strain was inoculated on plates containing various concentrations of the osmotic stress agent NaCl (Supplementary Figure S4). It was observed that in the presence of 0.5 M NaCl, the radial growth of all strains, including the wild type was not inhibited. At higher concentrations of NaCl (1 M and 1.5 M), the radial growth of all strains, including the wild type was significantly reduced. At 1.5 M NaCl, growth of all strains was minimal but overall, no differences were observed between strains with regards to the rates of vegetative growth in the presence of osmotic stress. The complementation of each gene resulted in similar phenotypes as those observed for the mutant strains and wild type (Supplementary Figure S5).

**FIGURE 3:**
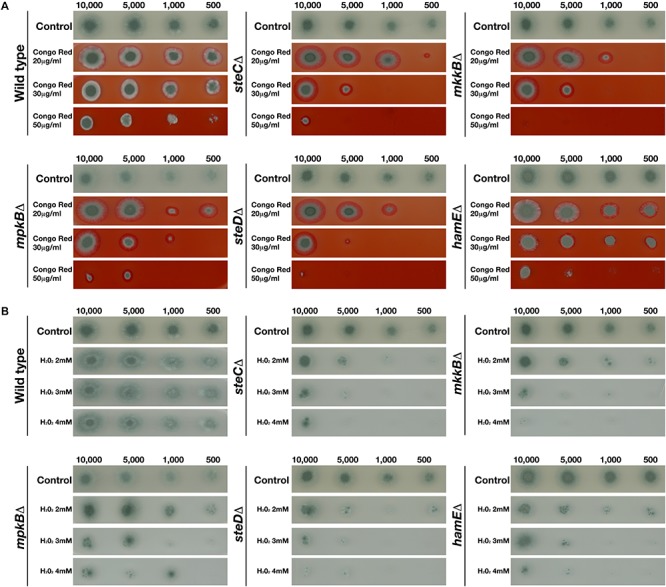
Growth phenotypes of mutant strains in the presence of various concentrations of Congo Red and H_2_O_2_. Strains were point-inoculated on GMM agar plates containing exogenous stress agents and left to incubate at 37°C for 3 days. The number of spores used for inoculation are listed above each panel. “Control” refers to strains point inoculated on GMM agar plates that did not contain any stress agents. **(A)** Growth phenotypes of the CEA17 wild-type strain and each mutant strain in the presence of 20, 30, and 50 μg Congo Red. **(B)** Growth phenotypes of the CEA17 wild-type strain and each mutant strain in the presence of 2, 3, or 4 mM H_2_O_2_.

To assess the influence of the pheromone module proteins in the response to cell wall stress specifically, each strain was inoculated on plates containing various concentrations (20, 30, and 50 μg/ml) of the cell wall stressor Congo Red (Figure 3A). It was observed that the CEA17 wild-type strain exhibited significant sensitivity to Congo Red at higher concentrations (50 μg/ml). However, it was evident that the deletion of *steC, mkkB, mpkB, steD*, and *hamE* resulted in increased sensitivity to all Congo Red concentrations tested. Each of these mutant strains displayed significant growth defects in the presence of each concentration of Congo Red, suggesting that these proteins may play a role in cell wall biosynthesis or maintenance. Complementation of each gene resulted in increased radial growth when compared to the respective mutants and the phenotypes of each complementation strain more closely resembled the wild-type phenotypes (Supplementary Figure S6).

To determine whether the pheromone module proteins contribute to the response to oxidative stress, each strain was inoculated on plates containing various concentrations (2, 3, and 4 mM) of the oxidative stress agent H_2_O_2_ (Figure 3B). It was observed that the CEA17 wild-type strain did not exhibit any significant growth impairments in the presence of any of the H_2_O_2_ concentrations tested. The radial growth of each inoculated CEA17 colony in the presence of H_2_O_2_ was similar to the growth on the control plates. However, it was observed that the wild-type colonies inoculated in the presence of H_2_O_2_ displayed significantly reduced sporulation levels in comparison to colonies on the control plates. For each of the mutant strains, it was apparent that the presence of H_2_O_2_ significantly impaired radial growth. The growth of each strain was reduced in the presence of all H_2_O_2_ concentrations tested and minimal growth was observed for each strain in the presence of 4mM H_2_O_2_. The complementation of each gene resulted in the restoration of radial growth, comparable to the rates observed for the wild-type colonies, albeit slightly smaller (Supplementary Figure S7). In the presence of each H_2_O_2_ concentration, these complementation strains closely resembled the wild-type colonies with regards to both radial growth and levels of sporulation.

Taken together, the results of these stress tests indicate that the pheromone module proteins contribute to the regulation of cellular responses to cell wall and oxidative stressors but do not influence sensitivity to osmotic stress. The deletion of any of the five members of the pheromone module results in increased sensitivity to both the cell wall stress agent Congo Red and the oxidative stressor H_2_O_2_.

### The Levels of Secondary Metabolite Production Are Reduced in Pheromone Module Mutant Strains

**FIGURE 4:**
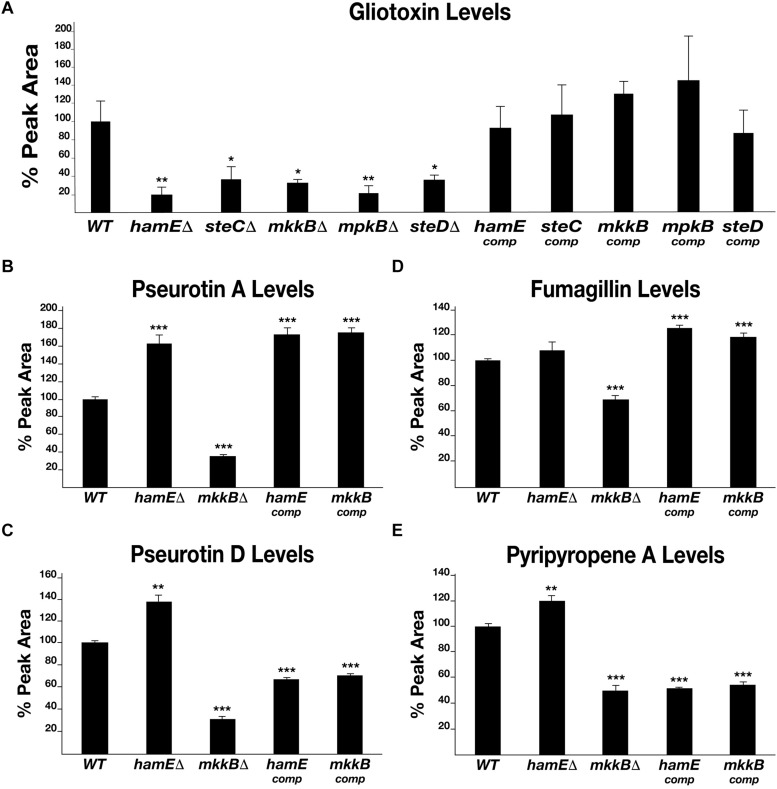
Levels of production of various metabolites in pheromone module mutant strains. **(A)** HPLC detection of gliotoxin levels in deletion and complementation strains. Each strain was inoculated (10^7^ spores/ml) in triplicate in 40 ml Czapek-Dox medium and left to incubate on a shaker at 37°C for 72 h. Average peak area values were plotted as a percentage of the wild type ± s.d. *P-*values were calculated by performing unpaired Student’s *t*-tests (*P < 0.05; **P < 0.01). For panels (B–E), strains were inoculated in triplicate in 40 ml of liquid GMM (5 million spores/ml) and incubated for 48 h at 37°C. Statistical calculations were performed as described for panel **(A)**. **(B)** Graphical representation of the pseurotin A levels in each strain. (**P < 0.01; ***P < 0.01). **(C)** Graphical representation of the pseurotin D levels in each strain. **(D)** Graphical representation of the fumagillin levels in each strain. **(E)**. Graphical representation of the pyripyropene A levels in each strain.

In order to determine the roles of the pheromone module proteins with regards to the regulation of secondary metabolism in *A. fumigatus*, the levels of various SMs produced by the pheromone module mutants were determined by LC-MS analysis (Figure 4 and Supplementary Table S9). *A. fumigatus* is capable of producing a myriad of SMs, many of which are uncharacterized. The most notable SM is the immunosuppressive agent gliotoxin, which is a major contributor to *A. fumigatus* virulence (Hof and Kupfahl, 2009; Ghazaei, 2017). Other notable SMs produced by *A. fumigatus* include (i) pseurotin A, a competitive inhibitor of chitin synthase which is also an inducer of nerve cell proliferation and acts as an immunosuppressive agent (Maiya et al., 2007; Ishikawa et al., 2009), (ii) pseurotin D, which exhibits apomorphine-antagonistic activity (Ishikawa and Ninomiya, 2008), (iii) fumagillin, an anti-angiogenic compound (Mc et al., 1951; Sin et al., 1997) and (iv) pyripyropene A, which exhibits insecticidal properties (Horikoshi et al., 2017).

By performing LC-MS analysis, the levels of each of the compounds listed above were determined in the pheromone module mutant strains and complementation strains. Gliotoxin was detected in all strains (Figure 4A). It was found that each mutant produces significantly less gliotoxin than the wild type. The average reductions in gliotoxin production for the mutant strains were between 63 and 80%. It was observed that the complementation of each gene restored the ability of these strains to produce gliotoxin to a level similar to that observed for the wild-type strain. The average levels of gliotoxin production for the complementation strains ranged between 87 and 145% of the wild-type average. Pseurotin A, pseurotin D, fumagillin and pyripyropene A were detected in the wild type, *hamE* mutant, *mkkB* mutant and respective complementation strains. Interestingly, the *hamE* mutant and the *mkkB* mutant exhibited different trends in production of all four metabolites tested. It was observed that pseurotin A production (Figure 4B) is increased in the *hamE* mutant (63% increase) and significantly decreased in the *mkkB* mutant (65% decrease). For pseurotin D (Figure 4C), the levels showed a similar trend, with an increase in production being observed in the *hamE* mutant (37% increase) and a significant decrease being evident in the *mkkB* mutant (69% decrease). Fumagillin production (Figure 4D) shows no significant difference between the wild type and *hamE* mutant, whereas in the *mkkB* mutant, the levels of production are dramatically reduced (31% decrease). Lastly, the levels of pyripyropene A (Figure 4E) were slightly increased in the *hamE* mutant (20% increase), whereas production of this compound was significantly decreased in the *mkkB* mutant (50% decrease).

Overall, these data suggest that MkkB is critical for the positive regulation of gliotoxin, pseurotin A, pseurotin D, fumagillin and pyripyropene A. However, HamE is required for the positive regulation of gliotoxin and negative regulation of pseurotin A, pseurotin D and pyripyropene A, whilst having no apparent effects in the regulation of fumagillin production. This could suggest that HamE may also act independently of the pheromone module to regulate secondary metabolism, perhaps in a similar manner to what is observed in *A. flavus* (Frawley et al., 2020).

### The Deletion of *mkkB* and *hamE* Does Not Reduce Virulence in a Murine Infection Model

To assess the influence of both *mkkB* and *hamE* in the regulation of *A. fumigatus* virulence, both mutant strains and the respective complementation strains were inoculated in mice to test the infectivity of these strains in a murine infection model of invasive aspergillosis (Supplementary Figure S8). When compared to the wild-type strain, it was evident that the deletion of both *mkkB* and *hamE* did not influence virulence, which complements the findings for *mpkB* (Manfiolli et al., 2019). This suggests that the pheromone module pathway is not required for the regulation of fungal virulence in murine infection models.

### The Localization of the Pheromone Module Is Cytoplasmic and MpkB Is the Only Protein That Translocates Into the Nucleus

**FIGURE 5:**
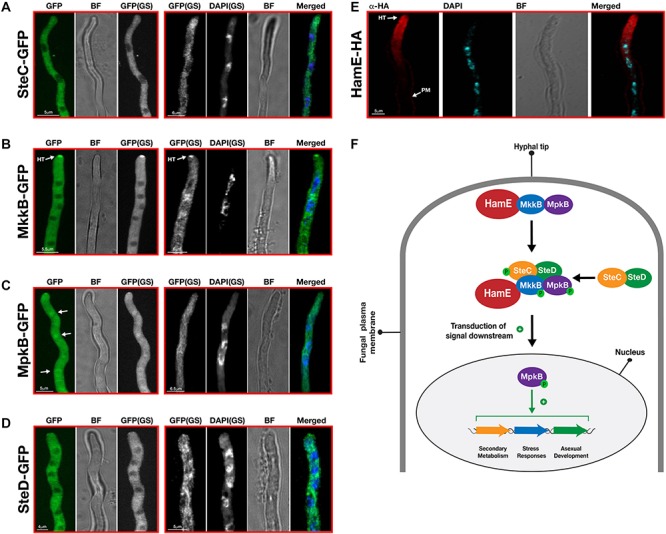
Sub-cellular localization patterns of the pheromone module proteins *in vivo.* **(A)** Sub-cellular localization of SteC-GFP. Strains from panels A–D were incubated at 30°C for various durations in 400 μL of liquid GMM, containing appropriate supplements. “BF” (brightfield images). “GS” (grayscale images). To visualize the nuclei, DAPI staining was performed. White arrows depict the accumulation of fusion protein in the nuclei. “HT” refers to accumulation of protein at hyphal tips. **(B)** Sub-cellular localization of MkkB-GFP. **(C)** Sub-cellular localization of MpkB-GFP. **(D)** Sub-cellular localization of SteD-GFP. **(E)** Sub-cellular localization of HamE-HA. The HamE-HA strain was inoculated (5 × 10^3^ spores) on sterile coverslips, covered in 450 μL of Sabouraud media, containing supplements. This strain were left to incubate at 30°C for 16 h. **(F)** Schematic model of the pheromone module in *A. fumigatus.* MkkB, MpkB, and HamE localize to the hyphal tips. These three proteins interact with the SteC-SteD dimer in the cytoplasm to form a pentameric complex which results in MpkB activation and transduction of a signal downstream to the nucleus. MpkB translocates into the nucleus, where it presumably interacts with transcription factors to positively regulate asexual sporulation, vegetative growth, stress responses and production of various SMs. “P” represents phosphate groups.

Confocal microscopy imaging was performed to determine the sub-cellular localizations of the pheromone module proteins *in vivo* (Figure 5). To monitor the localizations of these proteins in living material, strains were initially imaged without DAPI staining. To then compare the localizations of these proteins with respect to the nuclei, samples were subsequently fixed and stained with DAPI. Confocal microscopy imaging revealed that SteC-GFP displayed cytoplasmic fluorescence that was uniform throughout hyphae. This fusion protein was also shown to be excluded from interphase nuclei (Figure 5A). MkkB-GFP fluorescence was uniformly distributed throughout fungal hyphae. It was observed that this fusion protein is mostly cytoplasmic and is excluded from interphase nuclei and vacuoles. This protein was also observed to be enriched at the central portion of some septa and hyphal tips (Figure 5B). MpkB-GFP exhibited mostly uniform cytoplasmic distribution throughout fungal hyphae. However, MpkB was also observed to be slightly more concentrated in interphase nuclei and at the hyphal apices (Figure 5C). SteD-GFP fluorescence was faint and mostly uniform throughout the fungal hyphae. This fusion protein was found to be cytoplasmic and is excluded from interphase nuclei and vacuoles (Figure 5D). To observe the sub-cellular localization of HamE *in vivo*, immunostaining was performed, using the HamE-HA strain. It was observed that the HamE protein becomes enriched at the hyphal tips and the plasma membrane and is absent from interphase nuclei (Figure 5E).

Overall, these data complement findings in both *A. nidulans* (Bayram et al., 2012; Frawley et al., 2018) and *A. flavus* (Frawley et al., 2020), suggesting that a conserved mechanism of complex assembly and signaling exists in *A. fumigatus*. The kinases MkkB and MpkB may accumulate and interact with HamE at the hyphal tips, in response to pheromone signaling between neighboring hyphae. Signal detection could lead to the interaction of this trimer with the cytoplasmic SteC-SteD dimer to form a pentameric complex which enables efficient kinase phosphorylation and MpkB activation. Presumably, MpkB then translocates into the nucleus, where it interacts with various transcription factors to regulate a myriad of biological processes, such as vegetative growth, asexual sporulation, stress responses and secondary metabolism (Figure 5F). However, the exact molecular mechanism of signal transduction from hyphal tip to the nuclear envelope, as well as the direct targets of MpkB in the nucleus are currently unknown.

## Discussion

Eukaryotic organisms utilize a myriad of MAP kinase pathways to regulate a diverse range of biological processes (Schaeffer and Weber, 1999). The pheromone module in the model ascomycete fungus *A. nidulans* is a MAPK cascade that is activated in response to pheromone signaling, which occurs between neighboring hyphae and is critical for initiating hyphal fusion events (Bayram et al., 2012; Frawley et al., 2018). In this pathway, the three kinases SteC, MkkB, and MpkB are tethered to the membrane and hyphal tips via SteD adaptor interactions. MkkB and MpkB are bound by the HamE scaffold protein and this allows for efficient kinase phosphorylation in response to a detected stimulus. MpkB phosphorylation and translocation into the nucleus results in the activation of various transcription factors. Consequently, the pheromone module is involved in the regulation of asexual and sexual development, as well as secondary metabolism (Bayram et al., 2012; Frawley et al., 2018). The pheromone module is a highly conserved pathway that has been shown to exist in many organisms. For example, the homologous yeast Fus3 pathway is extensively studied and is responsible for promoting cell fusion and sexual development in response to pheromone signaling (Bardwell, 2005). The homologous MAK-2 pathway in the model filamentous fungus *Neurospora crassa* regulates germling and hyphal fusion (Pandey et al., 2004; Li et al., 2005). Recently, we have identified a homologous pheromone module in the plant and human pathogen *A. flavus.* This complex consists of the three kinases SteC, MkkB and MpkB, as well as the SteD adaptor. This pathway was shown to be critical for the regulation of asexual sporulation, sclerotia development and aflatoxin B1 production (Frawley et al., 2020).

Due to the high degree of conservation of the pheromone module, it was hypothesized that a similar mechanism of signaling could be utilized by the opportunistic human pathogen *A. fumigatus* to regulate development and secondary metabolism. This work highlights the identification of four homologous pheromone module proteins in *A. fumigatus* (SteC, MkkB, SteD and HamE). The *A. fumigatus* MpkB homolog has been previously identified and characterized (Manfiolli et al., 2019) but has not been studied within the context of the pheromone signaling pathway. In this study, we have shown, *via* a genetic and proteomic approach that these *A. fumigatus* homologous proteins physically interact to form a complex (Figures 1C,D and Supplementary Tables S4–S8), similar to what is observed in both *A. nidulans* (Bayram et al., 2012; Frawley et al., 2018) and *A. flavus* (Frawley et al., 2020). This complex could potentially consist of two sub-complexes. MkkB, MpkB and HamE have been shown to become enriched at the hyphal tips (Figures 5B,C,E, respectively) and thus, it is possible that they form a trimeric complex. Both SteC and SteD were dispersed throughout the fungal hyphae, indicating that they may form a dimer in the cytoplasm (Figures 5A,D, respectively). Interactions between these two sub-complexes could lead to assembly of the pentameric pheromone module in the cytoplasm, which complements the findings in *A. flavus* (Frawley et al., 2020). This would allow for MpkB activation and translocation into the nucleus, where it could interact with various transcription factors to modulate a diverse range of biological processes.

This work provides evidence that the pheromone module proteins are required for the positive regulation of both asexual sporulation and vegetative growth rate (Figure 2). All pheromone module mutants exhibited dramatically reduced sporulation levels (Figures 2A,C), as well as reduced colony diameters (Figure 2B). These data support the findings in *A. nidulans*, in which each mutant produced fewer conidia and the rates of hyphal growth were hindered in all mutants, aside from the *hamE* deletion strain (Frawley et al., 2018). These results do not fully duplicate the phenotypes observed in *A. flavus*, however, as the deletion of *hamE* in this species did not result in any defects in asexual sporulation or vegetative growth rate. Also, the deletion of *steC, mkkB, mpkB* or *steD* does not hinder the rates of hyphal growth in *A. flavus* (Frawley et al., 2020). Interestingly, in the study by Manfiolli et al. the deletion of *A. fumigatus mpkB* did not cause any defects in radial growth rate (Manfiolli et al., 2019). However, this mutant did exhibit significantly reduced levels of sporulation. The discrepancies in vegetative growth of the *mpkB* mutant in our study could be due to factors such as differences in the composition of the media used, the quantity of spores used for inoculation and the duration of incubation. Overall, our phenotypic data suggest that each of the pheromone module proteins contribute to the regulation of asexual development and hyphal growth in *A. fumigatus.* Due to the similarities in phenotypes observed for each mutant, these data provide evidence that each of these proteins function within the same pathway to regulate fungal development.

The influence of the pheromone module proteins in the regulation of various cell stress responses was also examined. Three main MAPK pathways become activated in *A. fumigatus* in response to stress. The CWI pathway is activated in response to cell wall stress agents and signals via the MAPK MpkA (Valiante et al., 2015; van de Veerdonk et al., 2017). The HOG pathway is required for the response to osmotic stressors and signals via the MAPK SakA (Du et al., 2006; Martinez-Montanes et al., 2010; de Nadal and Posas, 2015). Lastly, the HOG pathway, in cooperation with the MAPK MpkC have been shown to regulate cellular responses to osmotic, oxidative and cell wall stresses (Bruder Nascimento et al., 2016). It was observed that each pheromone module mutant strain exhibited significant defects in growth when cultured in the presence of exogenous stress agents such as the cell wall stressor Congo Red (Figure 3A) and the oxidative stressor H_2_O_2_ (Figure 3B). In relation to stress tests performed by Manfiolli et al., it was found that MpkB localization is increased in the nucleus in the presence of cell wall, oxidative and osmotic stress agents (Manfiolli et al., 2019). The *A. fumigatus mpkB* mutant also exhibited increased sensitivity to H_2_O_2_ as sporulation was drastically reduced in the presence of all H_2_O_2_ concentrations tested, which complements our findings. Interestingly, the presence of Congo Red did not impair vegetative growth of the wild type or the *mpkB* mutant. However, addition of the cell wall perturbing agent caspofungin caused significant reductions in the radial growth rates of this mutant. While this does not fully complement our findings, it does provide further evidence that MpkB plays a role in either the response to cell wall stressors or in cell wall biosynthesis. It appears that the strains used in the study by Manfiolli et al. (2019) exhibited increased resistance to Congo red as the growth of the wild type was not significantly impaired, even at 150 μg/ml concentration.However, in our study, a dramatic reduction in vegetative growth is evident for the wild type strain in the presence of 50 μg/ml Congo red. This could explain why the *mpkB* mutant in our study exhibits significantly reduced growth in the presence of Congo Red. Overall, these data provide evidence that the pheromone module proteins contribute to the regulation of cellular responses to stress, particularly cell wall and oxidative stress.

LC-MS analysis revealed that the pheromone module proteins are required for the regulation of SM production (Figure 4). *A. fumigatus* is a prolific producer of SMs, most notably the immunosuppressive agent gliotoxin (Hof and Kupfahl, 2009; Romsdahl and Wang, 2019). It was evident that the deletion of any of the five pheromone module genes results in dramatic reductions in gliotoxin production in comparison to a wild type strain (Figure 4A). It was also observed that the deletion of *mkkB* results in significantly reduced levels of pseurotin A, pseurotin D, fumagillin and pyripyropene A (Figures 4B–E). However, the deletion of *hamE* resulted in increased production of each of these compounds, with the exception of fumagillin, which showed no significant differences in comparison to the wild-type. Taken together, these data suggest that the pheromone module proteins contribute to the positive regulation of SM production, which complement the findings observed in both *A. nidulans* (Bayram et al., 2012; Frawley et al., 2018) and *A. flavus* (Frawley et al., 2020). However, these data also propose that HamE may exert regulatory roles that are independent of the pheromone module signalling pathway, which supports findings in *A. flavus* (Frawley et al., 2020).

In summary, this study has identified pheromone module homologs in the opportunistic human pathogen *A. fumigatus* and has provided evidence of the existence of a cytoplasmic pentameric complex, similar to what is observed in both *A. nidulans* (Bayram et al., 2012; Frawley et al., 2018) and *A. flavus* (Frawley et al., 2020). This complex consists of the three kinases SteC, MkkB and MpkB, as well as the SteD adaptor and the scaffold HamE, which together, enable transduction of a signal downstream and translocation of MpkB into the nucleus. MpkB would then presumably interact with transcription factors to regulate asexual sporulation, vegetative growth, stress responses and secondary metabolism (Figure 5F). According to the IP-MS data for MpkB-GFP (Supplementary Table S6), it was found that MpkB interacts with the SteA transcription factor, similar to what is observed in *A. nidulans* (Bayram et al., 2012). This transcription factor has been shown to be involved in the regulation of sexual development and hyphal fusion in various fungal species (Wong Sak Hoi and Dumas, 2010). MpkB was also found to interact with DvrA which is a C2H2 transcription factor. Orthologs of DvrA are predicted to play roles in the suppression of the host inflammatory response. Interestingly, MpkB was also found to interact with Afu1g04550. This is an uncharacterized protein in *A. fumigatus*, however, the ortholog of this protein in *A. nidulans* is HmbC. HmbC is a high mobility group box (HMGB) protein which is a chromatin-associated protein that has also been shown to interact with VeA, allowing for the regulation of development and secondary metabolism (Bokor et al., 2019). This could explain why MpkB in *A. fumigatus* was not found to interact directly with any of the velvet complex components, yet defects in both development and SM production were observed in the pheromone module mutant strains. However, despite the roles of the pheromone module in the regulation of both development and secondary metabolism, it was evident that this pathway is not essential for *A. fumigatus* virulence in a murine model, suggesting that compensatory mechanisms may also be utilized by this species to promote its virulence. These data also suggest that HamE may perform functions that are not associated with the pheromone module. Future studies will focus on characterizing the molecular roles of HamE to determine its functions within the context of the pheromone module signaling pathway as well as possible independent functions.

Overall, this work has provided insight on the molecular roles of the pheromone module in *A. fumigatus* and has contributed to the understanding of how MAPK signaling is utilized by filamentous fungal species to regulate their development and secondary metabolism in response to environmental stimuli.

## Data Availability Statement

All datasets generated for this study are included in the article/Supplementary Material.

## Ethics Statement

The animal study was reviewed and approved by Federal State authority: Thüringer Landesamt für Verbraucherschutz Ethics committee: Beratende Komission nach §15 Abs. 1 Tierschutzgesetz Permit number: 03-027/16.

## Author Contributions

Project conceptualisation, experimental design, data analysis and preparation of the manuscript was performed by DF. Preparation of samples for LC-MS analysis and formatting of results was performed by MCS. Confocal microscopy imaging was performed by BO. Murine infection models were performed by MS and TH. AF and AB were responsible for reviewing and editing the manuscript and provided resources for the experiments. ÖB was responsible for project conceptualisation, experimental design, supervision of the project, reviewing and editing the manuscript.

## Conflict of Interest

The authors declare that the research was conducted in the absence of any commercial or financial relationships that could be construed as a potential conflict of interest. The handling editor declared a past co-auhorship with one of the authors ÖB.

## Footnotes

1 http://www.aspgd.org

## References

[B1] Abad A. Fernandez-Molina J. V. Bikandi J. Ramirez A. Margareto J. Sendino J. (2010). What makes *Aspergillus fumigatus* a successful pathogen? Genes and molecules involved in invasive aspergillosis. *Rev. Iberoam. Micol.* 27 155–182. 10.1016/j.riam.2010.10.003 20974273

[B2] Altschul S. F. Gish W. Miller W. Myers E. W. Lipman D. J. (1990). Basic local alignment search tool. *J. Mol. Biol.* 215 403–410. 2231712 10.1016/S0022-2836(05)80360-2

[B3] Amare M. G. Keller N. P. (2014). Molecular mechanisms of *Aspergillus flavus* secondary metabolism and development. *Fungal Genet. Biol.* 66 11–18. 10.1016/j.fgb.2014.02.008 24613992

[B4] Atoui A. Bao D. Kaur N. Grayburn W. S. Calvo A. M. (2008). *Aspergillus nidulans* natural product biosynthesis is regulated by mpkB, a putative pheromone response mitogen-activated protein kinase. *Appl. Environ. Microbiol.* 74 3596–3600. 10.1128/AEM.02842-07 18378656 PMC2423048

[B5] Balloy V. Chignard M. (2009). The innate immune response to *Aspergillus fumigatus*. *Microb. Infect.* 11 919–927. 10.1016/j.micinf.2009.07.002 19615460

[B6] Bardwell L. (2005). A walk-through of the yeast mating pheromone response pathway. *Peptides* 26 339–350. 10.1016/j.peptides.2004.10.002 15690603 PMC3017506

[B7] Bayram O. Bayram O. S. Ahmed Y. L. Maruyama J. Valerius O. Rizzoli S. O. (2012). The *Aspergillus nidulans* MAPK module AnSte11-Ste50-Ste7-Fus3 controls development and secondary metabolism. *PLoS Genet.* 8:e1002816. 10.1371/journal.pgen.1002816 22829779 PMC3400554

[B8] Bayram O. Krappmann S. Ni M. Bok J. W. Helmstaedt K. Valerius O. (2008). VelB/VeA/LaeA complex coordinates light signal with fungal development and secondary metabolism. *Science* 320 1504–1506. 10.1126/science.1155888 18556559

[B9] Bokor E. Amon J. Keisham K. Karacsony Z. Vagvolgyi C. Hamari Z. (2019). HMGB proteins are required for sexual development in *Aspergillus nidulans*. *PLoS One* 14:e0216094. 10.1371/journal.pone.0216094 31022275 PMC6483251

[B10] Bruder Nascimento A. C. Dos Reis T. F. De Castro P. A. Hori J. I. Bom V. L. De Assis L. J. (2016). Mitogen activated protein kinases SakA(HOG1) and MpkC collaborate for *Aspergillus fumigatus* virulence. *Mol. Microbiol.* 100 841–859. 10.1111/mmi.13354 26878695

[B11] Dagenais T. R. Keller N. P. (2009). Pathogenesis of *Aspergillus fumigatus* in invasive aspergillosis. *Clin. Microbiol. Rev.* 22 447–465. 10.1128/CMR.00055-08 19597008 PMC2708386

[B12] de Castro E. Sigrist C. J. Gattiker A. Bulliard V. Langendijk-Genevaux P. S. Gasteiger E. (2006). ScanProsite: detection of PROSITE signature matches and ProRule-associated functional and structural residues in proteins. *Nucleic Acids Res.* 34 W362–W365. 10.1093/nar/gkl124 16845026 PMC1538847

[B13] de Nadal E. Posas F. (2015). Osmostress-induced gene expression–a model to understand how stress-activated protein kinases (SAPKs) regulate transcription. *FEBS J.* 282 3275–3285. 10.1111/febs.13323 25996081 PMC4744689

[B14] Du C. Sarfati J. Latge J. P. Calderone R. (2006). The role of the sakA (Hog1) and tcsB (sln1) genes in the oxidant adaptation of *Aspergillus fumigatus*. *Med. Mycol.* 44 211–218. 10.1080/13693780500338886 16702099

[B15] Elramli N. Karahoda B. Sarikaya-Bayram O. Frawley D. Ulas M. Oakley C. E. (2019). Assembly of a heptameric STRIPAK complex is required for coordination of light-dependent multicellular fungal development with secondary metabolism in *Aspergillus nidulans*. *PLoS Genet.* 15:e1008053. 10.1371/journal.pgen.1008053 30883543 PMC6438568

[B16] Frawley D. Greco C. Oakley B. Alhussain M. M. Fleming A. B. Keller N. P. (2020). The tetrameric pheromone module SteC-MkkB-MpkB-SteD regulates asexual sporulation, sclerotia formation and aflatoxin production in *Aspergillus flavus*. *Cell Microbiol.* e13192. 10.1111/cmi.13192 32068947 PMC7202998

[B17] Frawley D. Karahoda B. Sarikaya Bayram O. Bayram O. (2018). The HamE scaffold positively regulates MpkB phosphorylation to promote development and secondary metabolism in *Aspergillus nidulans*. *Sci. Rep.* 8:16588. 10.1038/s41598-018-34895-6 30410052 PMC6224500

[B18] Gardiner D. M. Waring P. Howlett B. J. (2005). The epipolythiodioxopiperazine (ETP) class of fungal toxins: distribution, mode of action, functions and biosynthesis. *Microbiology* 151 1021–1032. 10.1099/mic.0.27847-0 15817772

[B19] Ghazaei C. (2017). Molecular insights into pathogenesis and infection with *Aspergillus fumigatus*. *Malays. J. Med. Sci.* 24 10–20. 10.21315/mjms2017.24.1.2 28381925 PMC5345999

[B20] Hamel L. P. Nicole M. C. Duplessis S. Ellis B. E. (2012). Mitogen-activated protein kinase signaling in plant-interacting fungi: distinct messages from conserved messengers. *Plant Cell* 24 1327–1351. 10.1105/tpc.112.096156 22517321 PMC3398478

[B21] Hillmann F. Bagramyan K. Strassburger M. Heinekamp T. Hong T. B. Bzymek K. P. (2016). The crystal structure of peroxiredoxin Asp f3 provides mechanistic insight into oxidative stress resistance and virulence of *Aspergillus fumigatus*. *Sci. Rep.* 6:33396. 10.1038/srep33396 27624005 PMC5022050

[B22] Hof H. Kupfahl C. (2009). Gliotoxin in *Aspergillus fumigatus*: an example that mycotoxins are potential virulence factors. *Mycotoxin Res.* 25 123–131. 10.1007/s12550-009-0020-4 23605091

[B23] Horikoshi R. Goto K. Mitomi M. Oyama K. Sunazuka T. Omura S. (2017). Identification of pyripyropene A as a promising insecticidal compound in a microbial metabolite screening. *J. Antibiot. (Tokyo)* 70 272–276. 10.1038/ja.2016.155 28074053

[B24] Ishikawa M. Ninomiya T. (2008). Chemical modification of pseurotin A: one-pot synthesis of synerazol and pseurotin E and determination of absolute stereochemistry of pseurotin E. *J. Antibiot.* 61 692–695. 10.1038/ja.2008.99 19168986

[B25] Ishikawa M. Ninomiya T. Akabane H. Kushida N. Tsujiuchi G. Ohyama M. (2009). Pseurotin A and its analogues as inhibitors of immunoglobulin E [correction of immunoglobuline E] production. *Bioorg. Med. Chem. Lett.* 19 1457–1460. 10.1016/j.bmcl.2009.01.029 19179074

[B26] Latge J. P. (1999). *Aspergillus fumigatus* and aspergillosis. *Clin. Microbiol. Rev.* 12 310–350. 10194462 10.1128/cmr.12.2.310 PMC88920

[B27] Latge J. P. (2001). The pathobiology of *Aspergillus fumigatus*. *Trends Microbiol.* 9 382–389. 11514221 10.1016/s0966-842x(01)02104-7

[B28] Lewis L. Onsongo M. Njapau H. Schurz-Rogers H. Luber G. Kieszak S. (2005). Aflatoxin contamination of commercial maize products during an outbreak of acute aflatoxicosis in eastern and central Kenya. *Environ. Health Perspect.* 113 1763–1767. 10.1289/ehp.7998 16330360 PMC1314917

[B29] Li D. Bobrowicz P. Wilkinson H. H. Ebbole D. J. (2005). A mitogen-activated protein kinase pathway essential for mating and contributing to vegetative growth in *Neurospora crassa*. *Genetics* 170 1091–1104. 10.1534/genetics.104.036772 15802524 PMC1451179

[B30] Madeira F. Park Y. M. Lee J. Buso N. Gur T. Madhusoodanan N. (2019). The EMBL-EBI search and sequence analysis tools APIs in 2019. *Nucleic Acids Res.* 47 W636–W641. 10.1093/nar/gkz268 30976793 PMC6602479

[B31] Maiya S. Grundmann A. Li X. Li S. M. Turner G. (2007). Identification of a hybrid PKS/NRPS required for pseurotin A biosynthesis in the human pathogen *Aspergillus fumigatus*. *Chembiochem* 8 1736–1743. 10.1002/cbic.200700202 17722120

[B32] Manfiolli A. O. Siqueira F. S. Dos Reis T. F. Van Dijck P. Schrevens S. Hoefgen S. (2019). Mitogen-activated protein kinase cross-talk interaction modulates the production of melanins in *Aspergillus fumigatus*. *mBio* 10:e00215-19. 10.1128/mBio.00215-19 30914505 PMC6437049

[B33] Marshall C. J. (1994). MAP kinase kinase kinase, MAP kinase kinase and MAP kinase. *Curr. Opin. Genet. Dev.* 4 82–89. 8193545 10.1016/0959-437x(94)90095-7

[B34] Martinez-Montanes F. Pascual-Ahuir A. Proft M. (2010). Toward a genomic view of the gene expression program regulated by osmostress in yeast. *Omics* 14 619–627. 10.1089/omi.2010.0046 20726780

[B35] Maschmeyer G. Haas A. Cornely O. A. (2007). Invasive aspergillosis: epidemiology, diagnosis and management in immunocompromised patients. *Drugs* 67 1567–1601. 10.2165/00003495-200767110-00004 17661528

[B36] Mc C. M. Callender M. E. Lawlis J. F. Jr. (1951). Fumagillin (H-3), a new antibiotic with amebicidal properties. *Science* 113 202–203. 10.1126/science.113.2930.202 14809278

[B37] McCormick A. Loeffler J. Ebel F. (2010). *Aspergillus fumigatus*: contours of an opportunistic human pathogen. *Cell Microbiol.* 12 1535–1543. 10.1111/j.1462-5822.2010.01517.x 20716206

[B38] Mitchell A. L. Attwood T. K. Babbitt P. C. Blum M. Bork P. Bridge A. (2019). InterPro in 2019: improving coverage, classification and access to protein sequence annotations. *Nucleic Acids Res.* 47 D351–D360. 10.1093/nar/gky1100 30398656 PMC6323941

[B39] Pandey A. Roca M. G. Read N. D. Glass N. L. (2004). Role of a mitogen-activated protein kinase pathway during conidial germination and hyphal fusion in *Neurospora crassa*. *Eukaryot. Cell* 3 348–358. 10.1128/ec.3.2.348-358.2004 15075265 PMC387641

[B40] Rincon M. Davis R. J. (2009). Regulation of the immune response by stress-activated protein kinases. *Immunol. Rev.* 228 212–224. 10.1111/j.1600-065X.2008.00744.x 19290930

[B41] Rispail N. Soanes D. M. Ant C. Czajkowski R. Grunler A. Huguet R. (2009). Comparative genomics of MAP kinase and calcium-calcineurin signalling components in plant and human pathogenic fungi. *Fungal Genet. Biol.* 46 287–298. 10.1016/j.fgb.2009.01.002 19570501

[B42] Romsdahl J. Wang C. C. C. (2019). Recent advances in the genome mining of *Aspergillus* secondary metabolites (covering 2012-2018). *Medchemcomm* 10 840–866. 10.1039/c9md00054b 31303983 PMC6590338

[B43] Rushing B. R. Selim M. I. (2019). Aflatoxin B1: a review on metabolism, toxicity, occurrence in food, occupational exposure, and detoxification methods. *Food Chem. Toxicol.* 124 81–100. 10.1016/j.fct.2018.11.047 30468841

[B44] Saito H. (2010). Regulation of cross-talk in yeast MAPK signaling pathways. *Curr. Opin. Microbiol.* 13 677–683. 10.1016/j.mib.2010.09.001 20880736

[B45] Sarikaya Bayram O. Bayram O. Valerius O. Park H. S. Irniger S. Gerke J. (2010). LaeA control of velvet family regulatory proteins for light-dependent development and fungal cell-type specificity. *PLoS Genet.* 6:e1001226. 10.1371/journal.pgen.1001226 21152013 PMC2996326

[B46] Schaeffer H. J. Weber M. J. (1999). Mitogen-activated protein kinases: specific messages from ubiquitous messengers. *Mol. Cell. Biol.* 19 2435–2444. 10.1128/mcb.19.4.2435 10082509 PMC84036

[B47] Shaul Y. D. Seger R. (2007). The MEK/ERK cascade: from signaling specificity to diverse functions. *Biochim. Biophys. Acta* 1773 1213–1226. 10.1016/j.bbamcr.2006.10.005 17112607

[B48] Sin N. Meng L. Wang M. Q. Wen J. J. Bornmann W. G. Crews C. M. (1997). The anti-angiogenic agent fumagillin covalently binds and inhibits the methionine aminopeptidase. MetAP-2. *Proc. Natl. Acad. Sci. U.S.A.* 94 6099–6103. 10.1073/pnas.94.12.6099 9177176 PMC21008

[B49] Spikes S. Xu R. Nguyen C. K. Chamilos G. Kontoyiannis D. P. Jacobson R. H. (2008). Gliotoxin production in *Aspergillus fumigatus* contributes to host-specific differences in virulence. *J. Infect. Dis.* 197 479–486. 10.1086/525044 18199036

[B50] Valiante V. Macheleidt J. Foge M. Brakhage A. A. (2015). The *Aspergillus fumigatus* cell wall integrity signaling pathway: drug target, compensatory pathways, and virulence. *Front. Microbiol.* 6:325. 10.3389/fmicb.2015.00325 25932027 PMC4399325

[B51] Vallim M. A. Miller K. Y. Miller B. L. (2000). *Aspergillus* SteA (sterile12-like) is a homeodomain-C2/H2-Zn+2 finger transcription factor required for sexual reproduction. *Mol. Microbiol.* 36 290–301. 10.1046/j.1365-2958.2000.01874.x 10792717

[B52] van de Veerdonk F. L. Gresnigt M. S. Romani L. Netea M. G. Latge J. P. (2017). *Aspergillus fumigatus* morphology and dynamic host interactions. *Nat. Rev. Microbiol.* 15 661–674. 10.1038/nrmicro.2017.90 28919635

[B53] Widmann C. Gibson S. Jarpe M. B. Johnson G. L. (1999). Mitogen-activated protein kinase: conservation of a three-kinase module from yeast to human. *Physiol. Rev.* 79 143–180. 10.1152/physrev.1999.79.1.143 9922370

[B54] Wong Sak Hoi J. Dumas B. (2010). Ste12 and Ste12-like proteins, fungal transcription factors regulating development and pathogenicity. *Eukaryot. Cell* 9 480–485. 10.1128/EC.00333-09 20139240 PMC2863410

[B55] Yu J. Cleveland T. E. Nierman W. C. Bennett J. W. (2005). *Aspergillus flavus* genomics: gateway to human and animal health, food safety, and crop resistance to diseases. *Rev. Iberoam. Micol.* 22 194–202. 10.1016/s1130-1406(05)70043-7 16499411

