# Supplementary material for: The Pheromone Module SteC-MkkB-MpkB-SteD-HamE Regulates Development, Stress Responses and Secondary Metabolism in Aspergillus fumigatus
Source: Front Microbiol. 2020 May 7;11:811. doi: 10.3389/fmicb.2020.00811 (PMC7223695; doi:10.3389/fmicb.2020.00811)
Supplement: Supplementary file 1 [file Data_Sheet_1.docx]

**Supplementary Information**

**Supplementary methods**

**Strains and conditions for culturing**

Details of all strains used in this study are provided in Table S1. The *Aspergillus fumigatus* CEA17 (*pyrG*+) and CEA17 (*pyrG*Δ) strains served as wild type hosts for all deletions and epitope taggings. Various plasmids used for the knock-out, complementation and epitope tagging experiments are listed in Table S2. Plasmids were cloned into Stellar (Clontech) competent *Escherichia coli* cells and these cells were cultured in LB media (1% tryptone, 0.5% yeast extract, 1% NaCl), supplemented with 100μg/ml ampicillin and SOC media (2% tryptone, 0.5% yeast extract, 10mM NaCl, 2.5mM KCl, 10mM MgCl_2_, 10mM MgSO_4_, 20mM glucose).

For the growth of fungal strains on agar plates, Glucose Minimal Media (GMM) was used: (6g/L NaNO_3_, 0.52g/L KCl, 1.52g/L KH_2_PO_4_, 10g/L Glucose, 0.24g/L MgS0_4_, 0.1% trace element solution). Liquid media used were complete media (GMM media ingredients with the addition of 1g/L tryptone, 2g/L peptone and 1g/L yeast extract), Czapek-Dox media (233810 BD, 35g/L) and sabouraud media (CMO147 Oxoid, 30g/L). All appropriate supplements were added to media prior to inoculation with fungal spores. These supplements included uracil (1g/L), uridine (0.25g/L) and pyrithiamine (0.1mg/L). All fungal strains inoculated on agar plates and liquid media were cultured at 37^o^C. Strains inoculated in liquid media were left to incubate on a shaker at 180RPM for various durations.

**Plasmid Construction**

Details of all plasmids used in this study are given in Table S2 and all oligonucleotide sequences are provided in Table S3. The Lasergene Seqbuilder software was used to design all plasmid maps *in silico*. For all cloning experiments, pUC19 (Fermentas) digested with a *Sma*I restriction enzyme (Thermo Scientific), pOSB113 digested with a *Swa*I restriction enzyme (Thermo Scientific) and pSK379 digested with a *Pme*I restriction enzyme were used. For the construction of deletion fragments, 1.5-2 Kb 5’ UTR and 3’ UTR flanking regions of the gene of interest were PCR-amplified from genomic DNA with respective oligonucleotides. These UTR fragments were fused by fusion PCR to a selection marker (*pyrG, ptrA*) that was PCR-replicated from a plasmid containing the marker of interest. These three fragments were then cloned in the *Sma*I site of pUC19 by In-Fusion HD Cloning enzyme (Clontech, 121416) to create a circular plasmid.

To create the *sgfp* and *3xha* fusion constructs, 1.5-2 Kb of the gene ORF (with stop codon removed) and 1.5-2 Kb of the 3’ UTR sequences were PCR-amplified from WT genomic DNA and fused to either *sgfp* or *3xha* epitope tags connected to selection markers (*pyrG*). All epitope tags were fused to the gene ORFs at the C-terminal ends. These sets of three fragments were then cloned into the *Sma*I site of pUC19 and each ligated plasmid was transformed into competent bacterial cells.

To create the *hamE* complementation construct, primers were created to replicate a DNA fragment containing the entire *hamE* gene ORF and 1.5-2 Kb of both the 5’ and 3’ UTRs from a genomic DNA template. These genomic fragments were then cloned into the *Swa*I site of the pOSB113 plasmid. To create the *steC, mkkB, mpkB* and *steD* complementation constructs, the entire ORFs without UTR sequences were cloned into the *Pme*I site of the pSK379 plasmid, which contains a *ptrA* marker and a *gpdA* promoter. All ligated complementation plasmids were then transformed into competent bacterial cells. 10-15μg of each plasmid was transformed ectopically into fungal deletion recipient strains.

**Transformation of bacterial and fungal cells**

150μl of competent Stellar *E. coli* cells were added to ligated circular plasmid constructs on ice and left to incubate for 30 minutes. Samples were heat shocked at 42^o^C for 1 minute and immediately put back on ice. 800μl SOC media was added and samples were left to incubate on a shaker (180 RPM, 1 hour, 37^o^C). Samples were centrifuged for 1 minute (13,000 RPM), the supernatant was removed and bacterial cells were spread on LB agar plates containing ampicillin. Plates were left to incubate at 37^o^C for 16 hours.

For transformation of fungal cells, strains were cultured for 24 hours at 37^o^C in liquid complete media or sabouraud media. Mycelia were filtered through sterile miracloth and washed three times with citrate buffer (150mM KCl, 580mM NaCl, 50mM sodium citrate, pH5.5). Mycelia were transferred to sterile flasks and incubated in 20ml citrate buffer containing 500mg glucanase (Novozymes) and 240mg lysozyme (Serva) for 100 minutes at 30^o^C (50-60 RPM). Protoplasts were filtered through sterile miracloth into a 50ml falcon tube and pre-chilled STC1700 (1.2M sorbitol, 10mM Tris pH5.5, 50mM CaCl_2_, 35mM NaCl) was used to make the volume up to 50ml. Samples were inverted multiple times and left on ice for 10 minutes. Samples were centrifuged at 2,600 RPM for 15 minutes at 4^o^C. Supernatant was removed and pellet was resuspended in 1ml STC1700 buffer. The volume was made up to 50ml with STC1700 buffer and samples were centrifuged using the same settings. Supernatant was removed and pellet was resuspended in 1ml STC1700. Protoplasts were separated into two 150μl aliquots, with 1μg of linear DNA or 10-15μg of circular plasmid DNA being added to one and no DNA being added to the other, to act as a negative control. Samples were incubated on ice for 30 minutes. PEG4000 (60% PEG4000, 10mM Tris pH7.5, 50mM CaCl_2_) was added to each sample three times (2 x 250μl and 1 x 850μl aliquots). Following each addition, samples were inverted 15-20 times. Samples were left to incubate in a rack on top of ice for 30 minutes. STC1700 was added to each falcon to make the volume up to 15ml and samples were inverted. Falcon tubes were centrifuged at 2,600 RPM for 15 minutes at 4^o^C. Supernatant was discarded, the pellet was resuspended and protoplasts were inoculated on GMM agar plates containing 1.2M sorbitol and incubated at 37^o^C.

**Hybridization techniques**

The Lasergene SeqBuilder software was used to design the 5’ and 3’ UTR probes for hybridisation and allowed for selection of appropriate restriction enzymes for digesting genomic DNA. Probes were synthesised and labelled with Digoxigenin-11-UTP (PCR DIG Probe Synthesis Kit: Roche), using either 5’ or 3’ UTRs as templates and the respective oligonucleotides. Fungal genomic DNA was isolated from mycelia using the ‘Zymo Research Fungal/Bacterial Miniprep Kit’. 700ng was digested overnight with a suitable restriction enzyme and was separated on a 0.7% agarose gel at 100 Volts for 90 minutes. The gel was washed 3 times on a shaker at room temperature in the following solutions (0.25M HCl for 10 minutes, 0.5M NaOH/1.5M NaCl for 25 minutes and 1.5M NaCl/0.5M Tris for 30 minutes). The DNA fragments were transferred and UV cross-linked (UV Stratalinker 1800) to a nylon membrane (Amersham Hybond^TM^-N^+^, GE Healthcare). The membrane was washed twice with 2x SSC (Saline Sodium Citrate) buffer and dried for 5 minutes at 70^o^C. The membrane was incubated in a rotating tube at 42^o^C in pre-hybridisation buffer for 1 hour on a rotator. The probe was then added and left to incubate overnight.

The next day, the membrane was left on the rotator and was washed with 2x SSC/0.1% SDS solution for 5 minutes, followed by 2 washes with pre-heated 0.1x SSC/0.1% SDS for 20 minutes. The membrane was washed with 20ml DIG buffer 1, followed by incubation in 15ml DIG buffer 2 for 30 minutes. Alkaline phosphatase conjugated anti-DIG fab fragment (Roche 11093274910) was then added to the DIG buffer 2 (1:10,000 dilution) and left to incubate for 1 hour. 2 washes with 20ml wash buffer were performed for 15 minutes, followed by an incubation in 10ml DIG buffer 3 for 5 minutes. For chemiluminescent detection, CDP Star substrate (Roche) was added to the membrane and the membrane was exposed using the G:BOX Chemi XRQ (Syngene).

**Protein extraction and immunoprecipitation of fusion proteins**

Protein crude extracts were isolated from vegetative cultures that were incubated in liquid complete media on a shaker overnight. Mycelia were frozen using liquid nitrogen and pulverised with a mortar and pestle. Protein crude extracts were prepared by re-suspending the pulverised mycelia in 1ml protein extraction buffer (300mM NaCl, 50mM Tris-HCl pH 7.5, 10% glycerol, 1mM EDTA, 0.1% NP-40) that had been supplemented with 1mM DTT, 1X cOmplete EDTA-free protease inhibitors (Roche), 1mM benzamidine, 0.5mM PMSF and 1X phosphatase inhibitors (1mM NaF, 0.5mM sodium orthovanadate, 8mM β-glycerol phosphate) immediately prior to use. Samples were mixed vigorously by vortexing and centrifuged at 13,000 RPM for 10-15 minutes at 4^o^C. 1ml of the protein supernatant was transferred to a new 1.5ml microcentrifuge tube.

For the immunoprecipitation of GFP and HA fusion proteins, 10μl GFP-Trap sepharose (Chromotek) and 10μl anti-HA magnetic beads (Pierce) were washed twice with 190μl protein extraction buffer, containing supplements. The anti-GFP/HA beads were then resuspended in 50μl protein extraction buffer and added to 1ml crude protein extract. This mixture was left to incubate on a rotator for 3 hours at 4^o^C. Samples were placed in a magnetic rack and the supernatant was discarded. Beads were washed twice with 1ml protein extraction buffer (without supplements) and were then washed for a third time with the same buffer containing 1mM DTT. All liquid was removed and the beads were stored at -80^o^C until further use.

**Sample preparation for LC-MS/MS protein identification**

Isolated GFP and HA-tagged proteins were resuspended in 50mM ammonium bicarbonate. 1μl of 0.5M DTT was added and samples were incubated at 56^o^C for 20 minutes. 2.7μl of iodoacetamide (0.55M) was added and samples were incubated in the dark for 15 minutes. 1μl of 1% (w/v) ProteaseMAX (Promega) was added, followed by addition of 1μl trypsin (1μg/μl) (Promega). Samples were left to incubate overnight at 37^o^C. The next day, 1μl of Trifluoroacetic acid (TFA) was added to each and samples were vortexed briefly and left to incubate for 5 minutes at room temperature. Beads were collected on a magnetic rack and the supernatant was transferred to new tubes. The supernatants were centrifuged at 13,000 RCF for 10 minutes and dried in a SpeedVac for 3 hours. Samples were stored at -20^o^C until further use.

Peptide samples were resuspended in 20μl resuspension buffer (0.5% TFA) and sonicated for 3 minutes, followed by a brief centrifugation. ZipTip C_18_ pipette tips (Millipore) were used to purify peptide samples prior to mass spectrometric analysis. To equilibrate the ZipTips, a wetting solution (0.1%, 80% acetonitrile) was aspirated 5 times, followed by aspiration of an equilibration buffer (0.1% TFA) 5 times. ZipTips were then used to pipette the peptide samples up and down 15-20 times. Then, the equilibration buffer was aspirated again 5 times, followed by elution of the peptides via aspiration of an elution buffer (0.1% TFA, 60% acetonitrile) 5 times into a new E-cup. This solution was dried in a SpeedVac for 2 hours and peptide samples were stored at -20^o^C.

Immediately prior to loading, peptide samples were resuspended in 10μl Q-Exactive loading buffer (2% acetonitrile, 0.5% TFA) and 8μl was added to mass spectrometry vials (VWR). Samples were loaded on a high resolution quantitative LC-MS mass spectrometer (Thermo Fisher Q-Exactive). LC-MS identifications of peptides were performed using the Proteome Discoverer Daemon 1.4 software (Thermo Fisher) and organism-specific taxon-defined protein databases. Unique peptides were determined by isolating only those that do not appear in any of the wild type controls.

**Immunoblotting**

For all immunoblots, protean membranes (0.45μm pore size, GE Healthcare) were incubated in blocking solution [5% (w/v) non-fat dry milk solution in 1X TBS with 0.1% Tween 20] for 1 hour at room temperature with gentle shaking. For the detection of GFP-tagged proteins, mouse α-GFP antibody (SC-9996, SantaCruz) was used at 1:1,000 dilution in blocking solution for 2 hours at room temperature. Secondary goat α-mouse (170-6516, Biorad) was used at 1:2,000 dilution in blocking solution for 1 hour at room temperature. After each antibody incubation, membranes were washed three times with 1X TBST (0.1% Tween 20) for 5 minutes. For visualisation of all membranes, Luminata Crescendo Western HRP Substrate (Millipore) was added and membranes were exposed using the G:BOX Chemi XRQ (Syngene).

**RP-HPLC analysis of Gliotoxin levels**

To determine gliotoxin levels, *A. fumigatus* strains were inoculated (10^7^ spores/ml) in triplicate in 40ml czapek media and left to incubate on a shaker at 37^o^C for 72 hours at 180 RPM. The mycelia was filtered and 25ml of the supernatant was collected in falcon tubes and mixed with 25ml of chloroform, followed by incubation on a rotator at 4^o^C for 1 hour. Samples were centrifuged at 4,000 RPM for 15 minutes at 4^o^C. 20ml of the lower phase of each sample was transferred to new 50ml Falcon tubes and left to evaporate in a fume hood. Samples were resuspended in 2ml chloroform, transferred to 2ml microcentrifuge tubes and dried in a speedy-vac for 1 hour. Samples were resuspended in 200μl methanol and were loaded on a Shimadzu RP-HPLC with a photodiode array detector. 20μl of samples were injected onto a Luna**®**Omega 5μm Polar C18 (LC column 150 x 4.6m.m) and separated across a water:acetonitrile gradient with 0.1% (v/v) TFA. A gliotoxin standard (Sigma) of 10μg/ml was used as a reference. Gradient conditions of 5-100% acetonitrile over 30 minutes with a flow rate of 1ml/minute were used with a PDA detection at 254nm.

**Confocal Microscopy**

For confocal microscopy imaging, conidia were cultured in eight-chambered cover glasses (Lab-Tek; Thermo Fisher Scientific). Strains were incubated at 30 ^°^C for various durations in 400 μL of liquid GMM, containing appropriate supplements.

For 4′,6-diamidino-2-phenylindole (DAPI) staining experiments, germlings were initially fixed in the wells of the chambered cover glasses. To ensure fixation, the liquid medium was removed and 400 μL of fixative solution was added [8 % formaldehyde in 50 mM piperazine-N,N’-bis(2- ethanesulfonic acid) (PIPES), pH 6.7; 25 mM EGTA, pH 7.0; 5 mM MgSO4; and 5 % DMSO, pre-warmed to the culture temperature]. Samples were left to incubate for 30 min at 30 ^°^C. Following this incubation, the fixative solution was removed and replaced with 400 μL of 0.015 μg/mL DAPI solution.

To capture images, an UltraView VoX spinning disk confocal system (PerkinElmer) mounted on an Olympus IX71 inverted microscope was utilized. This confocal system is equipped with a piezoelectric stage which is software-controlled to enable rapid Z-axis movement. To collect images, a 60X/1.42 numerical aperture Olympus Plan Apo objective and an ORCA ERAG camera (Hamamatsu Photonics) were used. Solid state 405-nm and 488-nm lasers were used for excitation of DAPI and GFP respectively. For live imaging, the specimen temperature was maintained at 30 ^°^C using a temperature-controlled chamber. For DAPI experiments, fluorochrome-specific emission filters were used to prevent emission bleed through between fluorochromes. The system was controlled by Volocity software (PerkinElmer) running on a Power Mac computer (Apple). A stage micrometer was used to calibrate magnifications. After the adjustment of both minimum and maximum intensity levels (black and white levels) for each channel, the images were exported directly from Volocity.

**Immunostaining**

Coverslips were soaked in 100% ethanol for 10 seconds and flame sterilised. Sterile coverslips were then added to a 6-well macrotitre tray and 450μl of sabouraud media (containing supplements) was added to each coverslip. 5x10^3^ spores of each strain were used to inoculate the media and strains were left to incubate at 30^o^C for 14-16 hours.

The next day, fixation solution was freshly prepared by adding 0.6g paraformaldehyde to 15ml PME buffer (50mM PIPES, 25mM EGTA, 5mM MgSO_4_, pH adjusted to 6.7 with NaOH). The solution was left to incubate at 68^o^C for 45 minutes until paraformaldehyde had fully dissolved and then solution was left to cool to room temperature before use. All media was removed from wells by using a vacuum pump and 2ml of fixation solution was added to each coverslip. Samples were left to incubate at room temperature for 30 minutes. All fixation solution was removed using a vacuum pump and coverslips were washed three times for 5 minutes with 2ml PME buffer, with the liquid being removed after each wash. Lysing enzyme solution was prepared fresh by adding 400mg of lysing enzymes from *Trichoderma harzianum* (Sigma-L1412) to 20ml PME buffer. 10ml of this solution was added to 10ml egg white and 2ml of this solution was added to each coverslip. Samples were left to incubate at 25^o^C for 50 minutes while shaking slowly (50 RPM). Lysing enzyme solution was then removed and samples were washed three times for 10 minutes with 2ml PME buffer. 1.5ml extraction buffer (100mM PIPES, 25mM EGTA, 0.1% NP-40) was added to each coverslip and samples were left to incubate for 9 minutes at room temperature. Liquid was removed and samples were washed once with 1.5ml PME buffer. 1.5ml of ice-cold methanol was then added to each coverslip and samples were left to incubate for 10 minutes at room temperature. Samples were then washed twice with 1ml PME buffer for 5 minutes. 2ml of TBST (3% BSA) solution was added to each coverslip and samples were left to incubate for 30 minutes at room temperature.

200μl of primary antibody solutions (1:50 mouse α-GFP, 1:100 mouse α-HA) were added to the coverslips and samples were left to incubate for 1 hour at room temperature. Samples were then washed three times with 250μl TBST for 5 minutes. 200μl of secondary antibody solution (1:100 goat anti-mouse Alexa fluor 594-Abcam:ab150120) was added to each coverslip and samples were left to incubate at room temperature for 1 hour in the dark. Samples were washed with 250μl TBST 3 times for 5 minutes. All liquid was removed and one drop of mounting medium (ProLong gold antifade mountant with DAPI-ThermoFisher Scientific:P36941) was added to a microscope slide. Each coverslip was placed germling side down on the microscope slide and the excess mounting medium was removed using filter paper. Nail polish was added to the microscope slides and was left to dry for 10 minutes at room temperature. Samples were stored overnight at 4^o^C in the dark. Localisations of proteins were detected using the Olympus FluoView1000 laser scanning confocal microscope.

**Protein domain searches**

Detection of protein sizes and domains were performed using a combination of ScanProsite ^1^ and InterPro^2^ softwares. Detection of protein homologs was performed by reciprocal BLAST searches ^3^

**Murine infection models**

Specific-pathogen-free female outbreed CD-1 mice (18-20g, 8-10 week-old) were supplied by Charles River, Germany. Animals were housed under standard conditions in individually ventilated cages and fed with normal mouse chow and water *ad libitum*. All animals were cared for in accordance with the European animal welfare regulation and approved by the responsible state authority and ethics committee in accordance with the German animal welfare act. Mice were immunosuppressed with two single doses of 25 mg cortisone acetate (Sigma-Aldrich), which were injected intraperitoneally three days before and 2 hours prior to infection (day 0). Mice were anesthetized by an intraperitoneal anesthetic combination of midazolam, fentanyl, and medetomidine. Mice were infected intranasally with 2.5x10^5^ conidia in 25 µl PBS (10 mice/strain; 4 mice for PBS control). Anesthesia was terminated by subcutaneous injection of flumazenil, naloxon and atipamezol. Infected animals were monitored twice daily and humanely sacrificed if moribund (defined by lethargy, dyspnea, hypothermia and weight loss).

**Supplementary Figures**

**
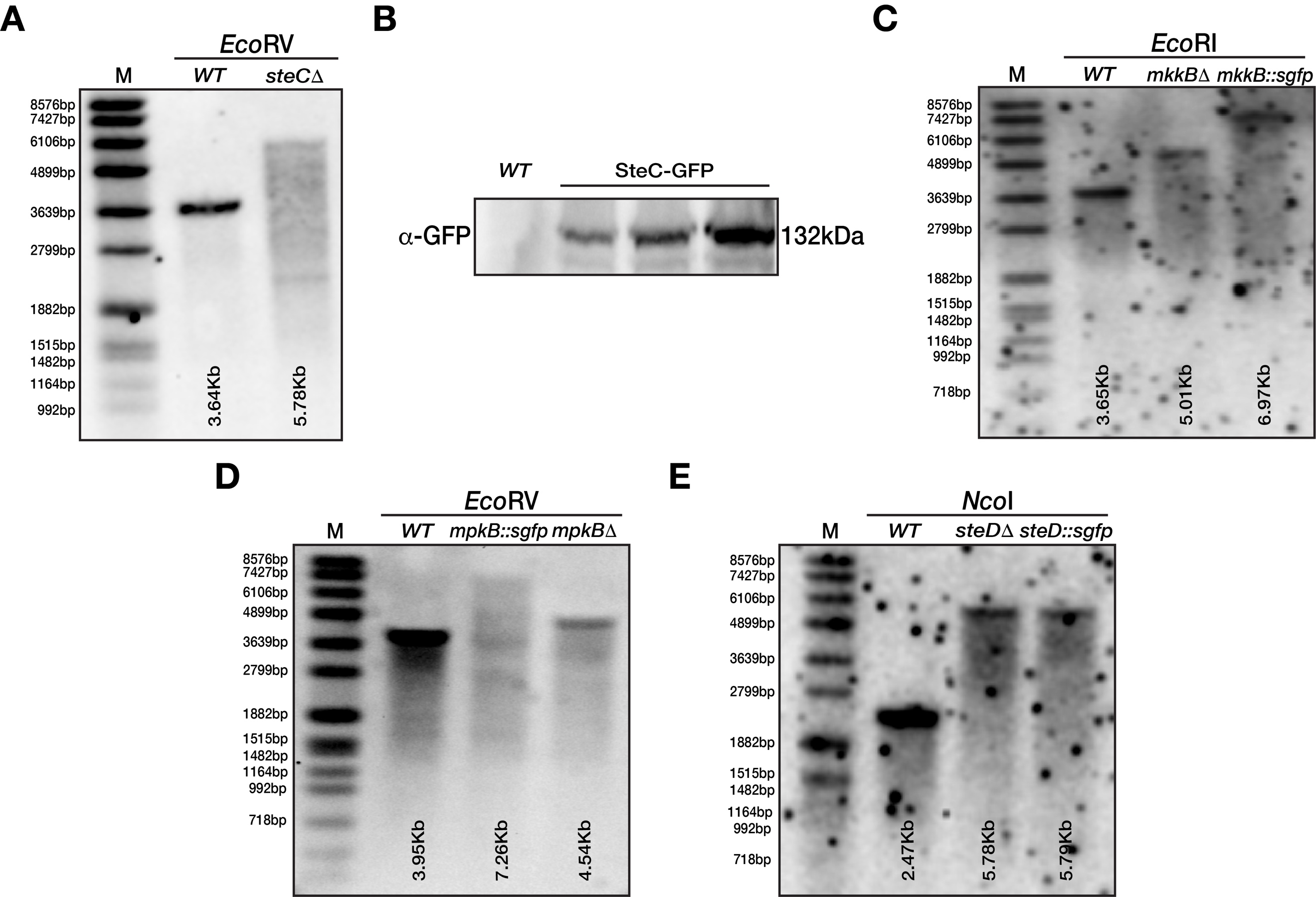
**

**Figure S1. Confirmation of deletions and tagged *A. fumigatus* strains via southern blotting** (A) Southern hybridizations of *steC*Δ. M: Molecular marker in basepairs (bp). Sizes of the bands shown for the wild type CEA17 strain and the deletion strain are in accordance with theoretical maps. The *Eco*RV restriction enzyme was used to digest genomic DNA and a 3’ UTR DIG-labelled probe was used for detection. (B) Western blot detecting the presence of the functional SteC-GFP fusion protein in three *A. fumigatus* clones via an α-GFP antibody. The size of the tagged protein is 132kDa as predicted. (C) Southern hybridizations of *mkkB*Δ and *mkkB::sgfp*. The *Eco*RI restriction enzyme was used to digest genomic DNA and a 3’ UTR DIG-labelled probe was used for detection. (D) Southern hybridizations of *mpkB*Δ and *mpkB::sgfp.* The *Eco*RV restriction enzyme was used to digest genomic DNA and a 5’ UTR DIG-labelled probe was used for detection. (E) Southern hybridizations of *steD*Δ and *steD::sgfp.* The *Nco*I restriction enzyme was used to digest genomic DNA and a 3’ UTR DIG-labelled probe was used for detection.

**Figure S2: Confirmation of *hamE* deletions and tagged *hamE* strains via southern blotting.** Southern hybridizations of two *hamE* deletion, *hamE::sgfp* and *hamE::3xha* clones. M: Molecular marker in basepairs (bp). Sizes of the bands shown for each strain are in accordance with theoretical maps. The *Sca*I restriction enzyme was used to digest genomic DNA and a 3’ UTR probe was used for detection.


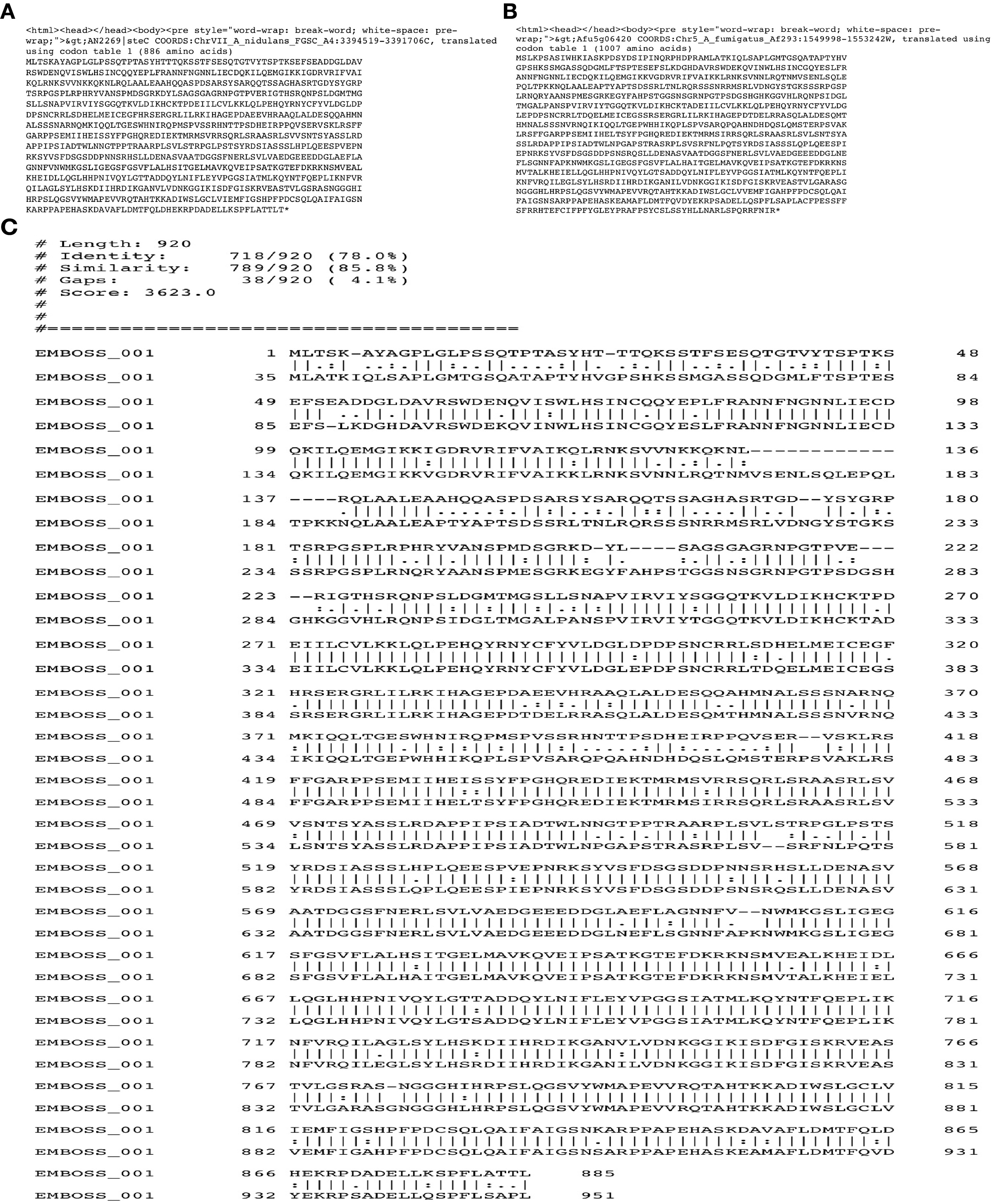


**Figure S3: Alignment of *A. nidulans* SteC (AN2269) and *A. fumigatus* SteC (Afu5g06420) protein sequences.** (A) Protein sequence of *A. nidulans* SteC (AN2269). (B) Protein sequence of *A. fumigatus* SteC (Afu5g06420). (C) Pairwise sequence alignment of both SteC protein sequences using the Smith-Waterman algorithm ^4^.

**
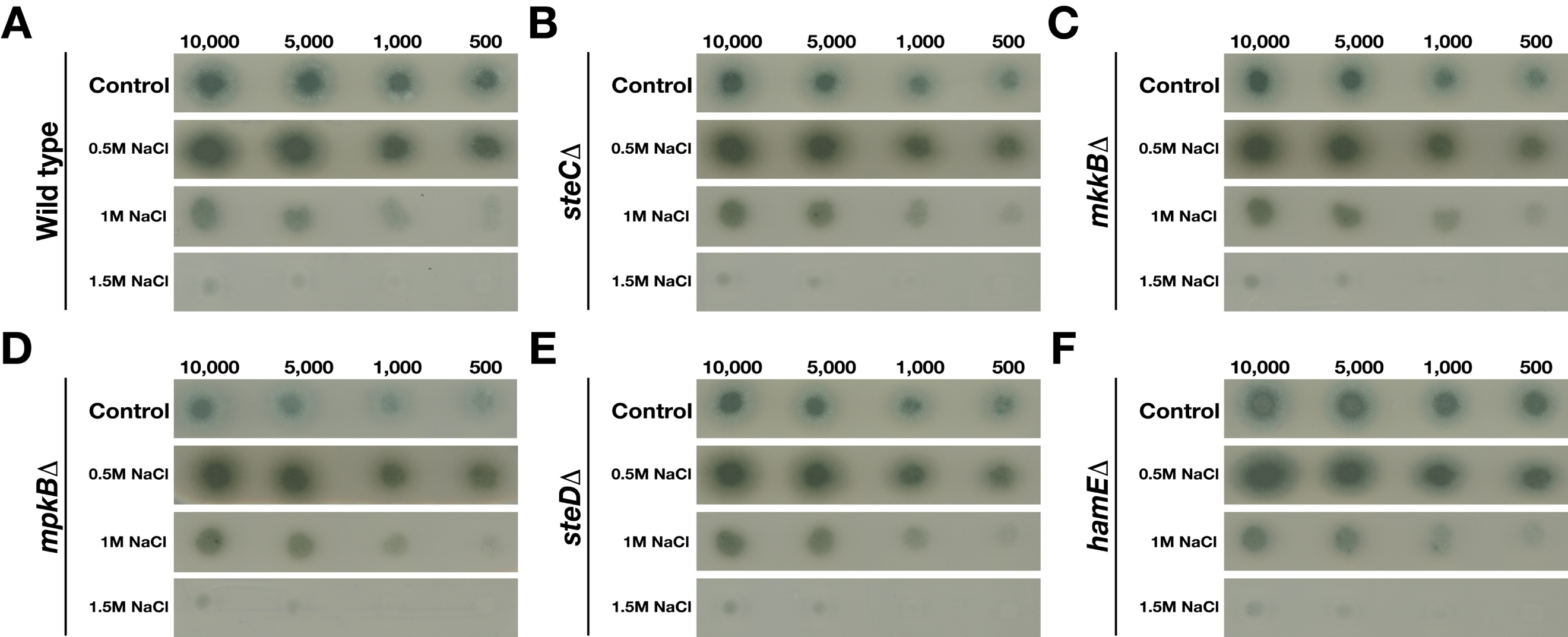
**

**Figure S4: Growth phenotypes of mutant strains in the presence of various concentrations of NaCl.** Strains were point-inoculated on GMM agar plates containing either 0.5M, 1M or 1.5M NaCl and left to incubate at 37°C for 3 days. The number of spores used for inoculation are listed above each panel. ‘Control’ refers to strains point inoculated on GMM agar plates that did not contain any stress agents. (A) CEA17 wild type strain phenotypes. (B) *steC* mutant strain phenotypes. (C) *mkkB* mutant strain phenotypes. (D) *mpkB* mutant strain phenotypes. (E) *steD* mutant strain phenotypes. (F) *hamE* mutant strain phenotypes.

**
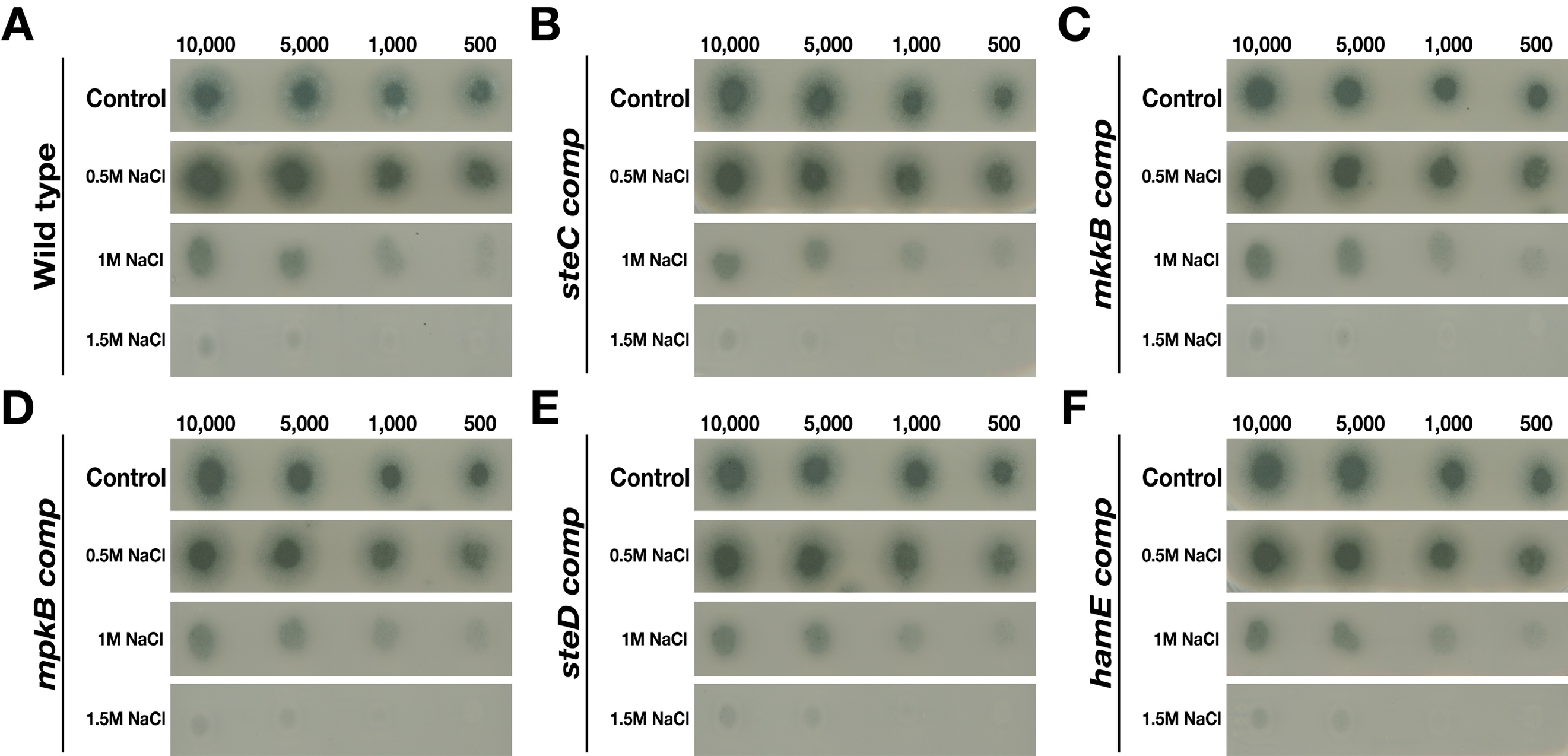
**

**Figure S5: Growth phenotypes of complementation strains in the presence of various concentrations of NaCl.** Strains were point-inoculated on GMM agar plates containing either 0.5M, 1M or 1.5M NaCl and left to incubate at 37^o^C for 3 days. The number of spores used for inoculation are listed above each panel. ‘Control’ refers to strains point inoculated on GMM agar plates that did not contain any stress agents. (A) CEA17 wild type strain phenotypes. (B) *steC* complementation strain phenotypes. (C) *mkkB* complementation strain phenotypes. (D) *mpkB* complementation strain phenotypes. (E) *steD* complementation strain phenotypes. (F) *hamE* complementation strain phenotypes.

**
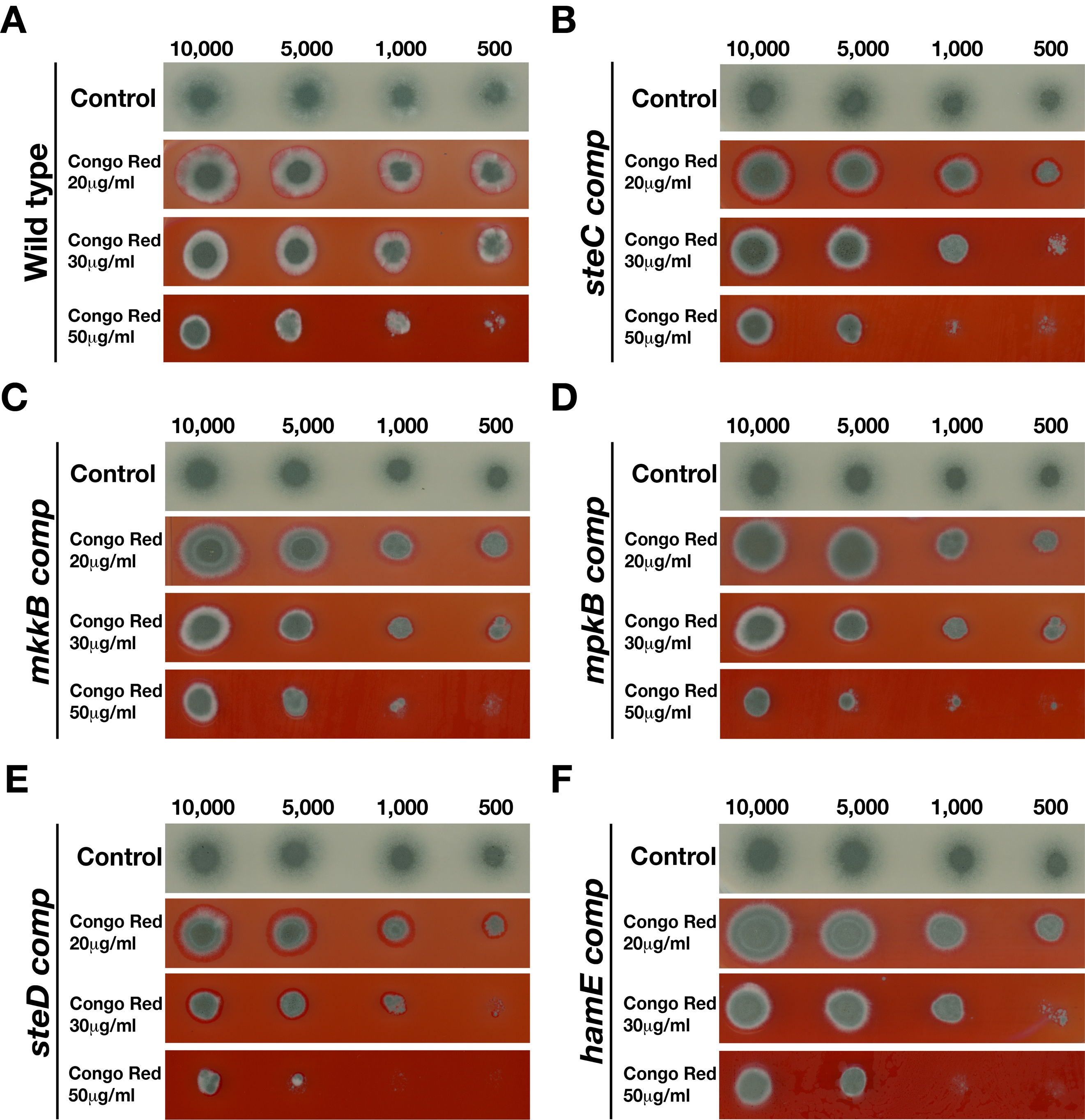
**

**Figure S6: Growth phenotypes of complementation strains in the presence of various concentrations of congo red.** Strains were point-inoculated on GMM agar plates containing either 20μg, 30μg or 50μg congo red and left to incubate at 37^o^C for 3 days. The number of spores used for inoculation are listed above each panel. ‘Control’ refers to strains point inoculated on GMM agar plates that did not contain any stress agents. (A) CEA17 wild type strain phenotypes. (B) *steC* complementation strain phenotypes. (C) *mkkB* complementation strain phenotypes. (D) *mpkB* complementation strain phenotypes. (E) *steD* complementation strain phenotypes. (F) *hamE* complementation strain phenotypes.

**
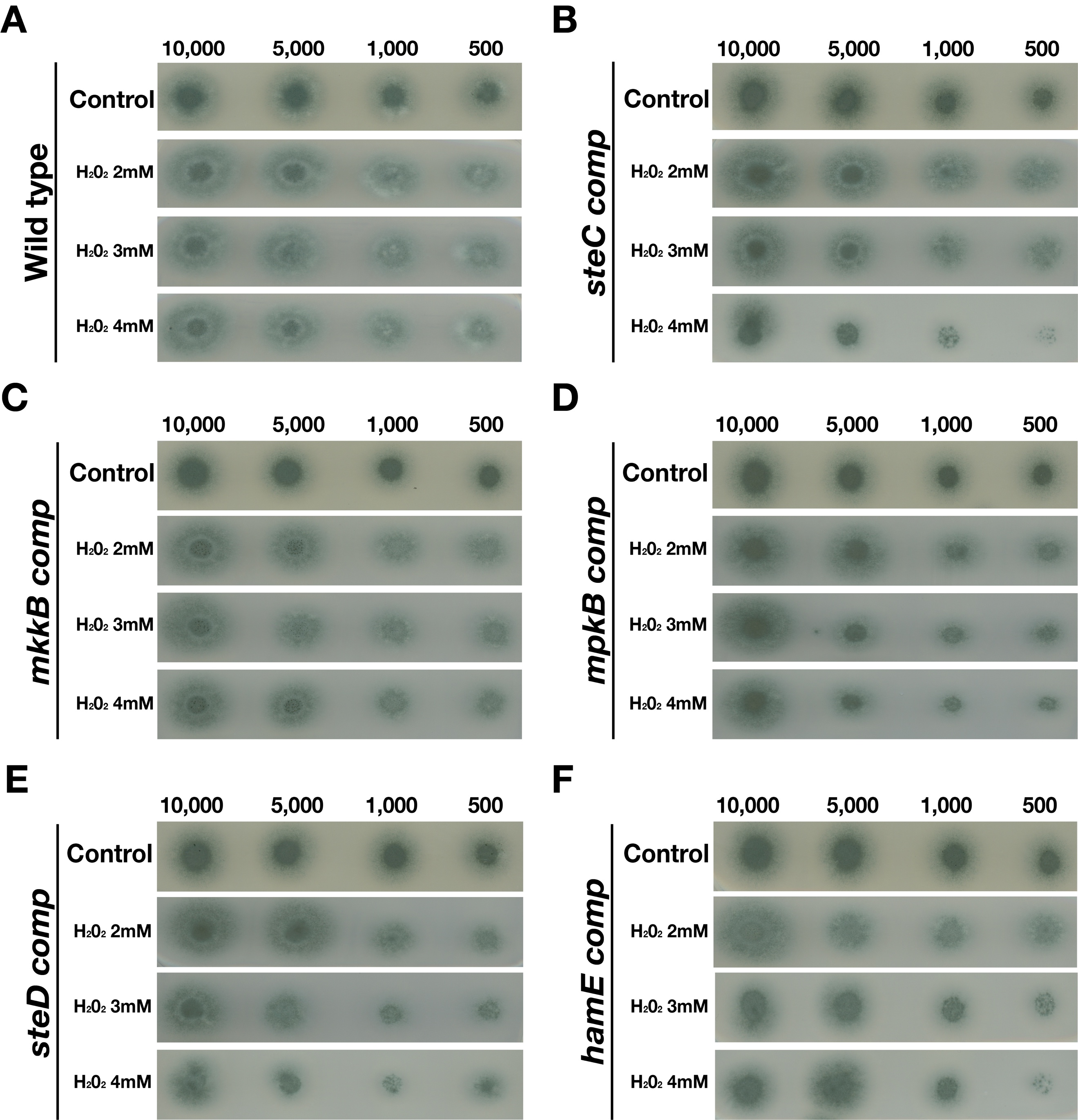
**

**Figure S7: Growth phenotypes of complementation strains in the presence of various concentrations of H_2_0_2_.** Strains were point-inoculated on GMM agar plates containing either 2mM, 3mM or 4mM H_2_0_2_ and left to incubate at 37^o^C for 3 days. The number of spores used for inoculation are listed above each panel. ‘Control’ refers to strains point inoculated on GMM agar plates that did not contain any stress agents. (A) CEA17 wild type strain phenotypes. (B) *steC* complementation strain phenotypes. (C) *mkkB* complementation strain phenotypes. (D) *mpkB* complementation strain phenotypes. (E) *steD* complementation strain phenotypes. (F) *hamE* complementation strain phenotypes.


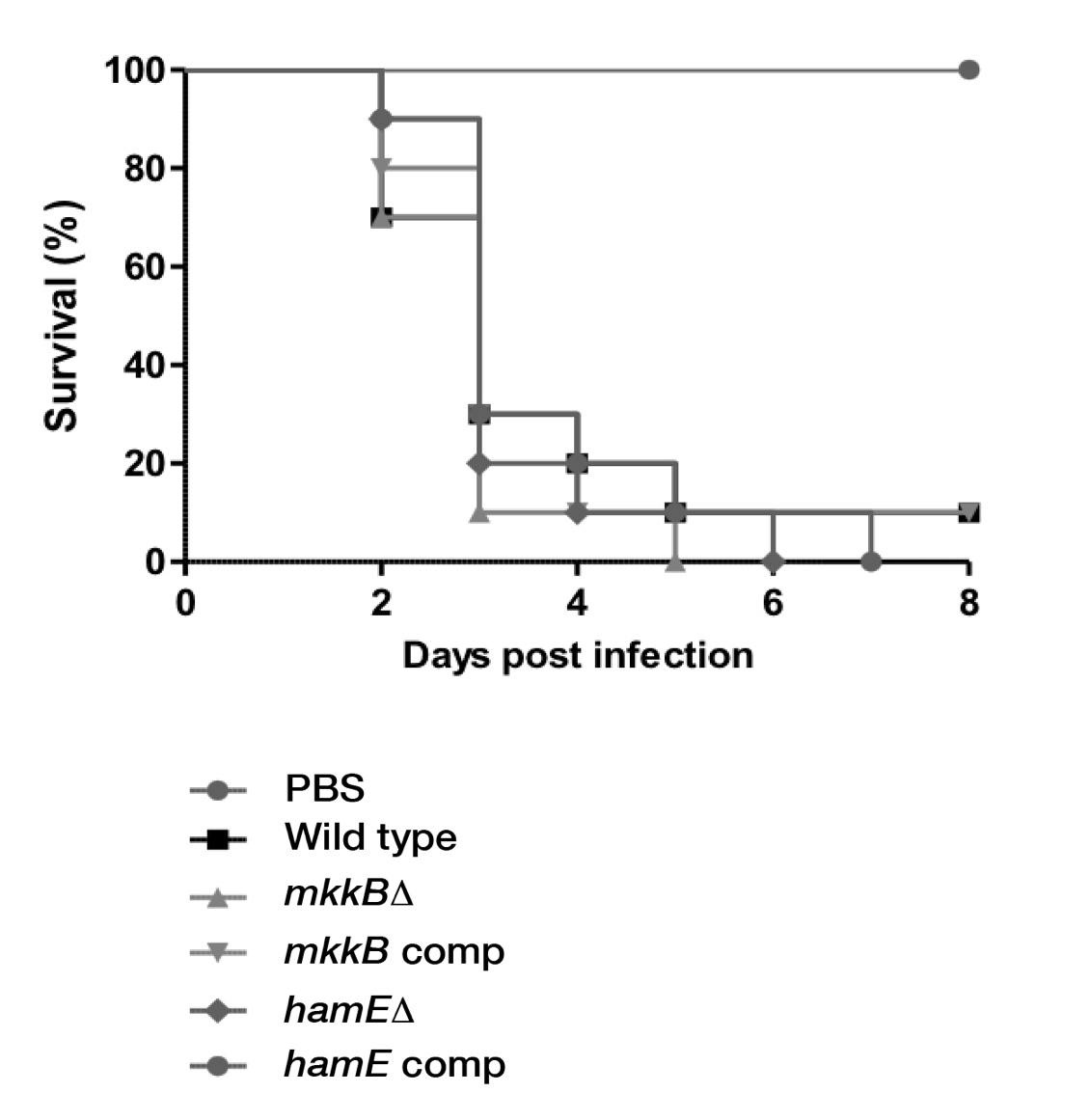


**Figure S8: Rates of survival of mice infected with *A fumigatus* conidia.** Specific pathogen-free female outbreed CD-1 mice were infected in groups of 10 intranasally with *A. fumigatus* conidia (2.5x10^5^ conidia in 25μl PBS). Fungal strains used for infection were the CEA17 wild type, *mkkB* mutant, *mkkB* complementation, *hamE* mutant and *hamE* complementation strain. 4 mice were infected with PBS to serve as a control.

**Supplementary References**

1 de Castro, E. *et al.* ScanProsite: detection of PROSITE signature matches and ProRule-associated functional and structural residues in proteins. *Nucleic Acids Res* **34**, W362-365, doi:10.1093/nar/gkl124 (2006).

2 Mitchell, A. L. *et al.* InterPro in 2019: improving coverage, classification and access to protein sequence annotations. *Nucleic Acids Res* **47**, D351-d360, doi:10.1093/nar/gky1100 (2019).

3 Altschul, S. F., Gish, W., Miller, W., Myers, E. W. & Lipman, D. J. Basic local alignment search tool. *J Mol Biol* **215**, 403-410, doi:10.1016/s0022-2836(05)80360-2 (1990).

4 Madeira, F. *et al.* The EMBL-EBI search and sequence analysis tools APIs in 2019.

5 da Silva Ferreira, M. E. *et al.* The akuB(KU80) mutant deficient for nonhomologous end joining is a powerful tool for analyzing pathogenicity in *Aspergillus fumigatus*. *Eukaryot Cell* **5**, 207-211, doi:10.1128/ec.5.1.207-211.2006 (2006).

6 Szewczyk, E. & Krappmann, S. Conserved regulators of mating are essential for *Aspergillus fumigatus* cleistothecium formation. *Eukaryot Cell* **9**, 774-783, doi:10.1128/ec.00375-09 (2010).
