# Supplementary material for: The Pheromone Module SteC-MkkB-MpkB-SteD-HamE Regulates Development, Stress Responses and Secondary Metabolism in Aspergillus fumigatus
Source: Front Microbiol. 2020 May 7;11:811. doi: 10.3389/fmicb.2020.00811 (PMC7223695; doi:10.3389/fmicb.2020.00811)
Supplement: Supplementary file 2 [file Table_1.DOCX]

**Supplementary Table S1:** Fungal strains created or used in this study

| **Strain** | **Genotype** | **Plasmid used** | **Reference** |
| --- | --- | --- | --- |
| CEA17 | *Wild type, akuB∆::pyrG, MAT1-1* | Not applied | **^5^** |
| CEA17 (*pyrG∆*) | *Wild type, akuB∆, pyrG∆, MAT1-1* | Not applied | Jean-Paul Latge Lab |
| AFUDF1 | *hamE∆::ptrA, akuB∆, pyrG+, MAT1-1* | pDF4 in CEA17 | This Study |
| AFUDF2 | *hamE::sgfp::hph, akuB∆, pyrG+, MAT1-1* | pDF5 in CEA17 | This Study |
| AFUDF3 | *hamE::3xha::hph, akuB∆, pyrG+, MAT1-1* | pDF6 in CEA17 | This Study |
| AFUDF12 | *steC∆::pyrG, akuB∆, pyrG∆, MAT1-1* | pDF22 in CEA17 (*pyrG*∆) | This Study |
| AFUDF13 | *mkkB∆::pyrG, akuB∆, pyrG∆, MAT1-1* | pDF23 in CEA17 (*pyrG*∆) | This Study |
| AFUDF14 | *mpkB∆::pyrG, akuB∆, pyrG∆, MAT1-1* | pDF24 in CEA17 (*pyrG*∆) | This Study |
| AFUDF15 | *steD∆::pyrG, akuB∆, pyrG∆, MAT1-1* | pDF25 in CEA17 (*pyrG*∆) | This Study |
| AFUDF17 | *mkkB::sgfp::pyrG, akuB∆, pyrG∆, MAT1-1* | pDF27 in CEA17 (*pyrG*∆) | This Study |
| AFUDF18 | *mpkB::sgfp::pyrG, akuB∆, pyrG∆, MAT1-1* | pDF28 in CEA17 (*pyrG*∆) | This Study |
| AFUDF19 | *steD::sgfp::pyrG, akuB∆, pyrG∆, MAT1-1* | pDF29 in CEA17 (*pyrG*∆) | This Study |
| AFUDF20 | *hamE∆::ptrA, akuB∆, pyrG∆, MAT1-1* | pDF4 in CEA17 (*pyrG*∆) | This Study |
| AFUDF26 | *gpdA::steC::ptrA, steC∆::pyrG, akuB∆, pyrG∆, MAT1-1* | pDF44 in AFUDF12 | This Study |
| AFUDF27 | *gpdA::mkkB::ptrA, mkkB∆::pyrG, akuB∆, pyrG∆, MAT1-1* | pDF45 in AFUDF13 | This Study |
| AFUDF28 | *gpdA::mpkB::ptrA, mpkB∆::pyrG, akuB∆, pyrG∆, MAT1-1* | pDF46 in AFUDF14 | This Study |
| AFUDF29 | *gpdA::steD::ptrA, steD∆::pyrG, akuB∆, pyrG∆, MAT1-1* | pDF47 in AFUDF15 | This Study |
| AFUDF31 | *_p_hamE::hamE::hamE_t_::pyrG, hamE∆::ptrA, pyrG-, MAT1-1* | pDF49 in AFUDF20 | This Study |
| AFUDF33 | *steC::sgfp::pyrG, akuB∆, pyrG∆, MAT1-1* | pDF55 in CEA17 (*pyrG*∆) | This Study |
