# Supplementary material for: The Pheromone Module SteC-MkkB-MpkB-SteD-HamE Regulates Development, Stress Responses and Secondary Metabolism in Aspergillus fumigatus
Source: Front Microbiol. 2020 May 7;11:811. doi: 10.3389/fmicb.2020.00811 (PMC7223695; doi:10.3389/fmicb.2020.00811)
Supplement: Supplementary file 3 [file Table_2.DOCX]

**Supplementary Table S2:** Plasmids created or used in this study

| **Plasmid** | **Description** | **Reference** |
| --- | --- | --- |
| pUC19 | *E. coli* cloning plasmid with *bla* (ampicillin resistance) gene | Thermo Fisher |
| pOSB113 | *Pme*I*::AfpyrG::Swa*I inserted in *Sma*I site of pUC19 | This Study |
| pSK379 | *^p^gpdA-his2A^t^* and *ptrA* resistance cassette | **^6^** |
| pDF4 | *hamE* deletion with *ptrA* in *Sma*I site of pUC19 | This Study |
| pDF5 | *hamE:*:*sgfp::hph* cassette in *Sma*I site of pUC19 | This Study |
| pDF6 | *hamE:*:*3xha::hph* cassette in *Sma*I site of pUC19 | This Study |
| pDF22 | *steC* deletion with *pyrG* in *Sma*I site of pUC19 | This Study |
| pDF23 | *mkkB* deletion with *pyrG* in *Sma*I site of pUC19 | This Study |
| pDF24 | *mpkB* deletion with *pyrG* in *Sma*I site of pUC19 | This Study |
| pDF25 | *steD* deletion with *pyrG* in *Sma*I site of pUC19 | This Study |
| pDF27 | *mkkB::sgfp::pyrG* in *Sma*I site of pUC19 | This Study |
| pDF28 | *mpkB::sgfp::pyrG* in *Sma*I site of pUC19 | This Study |
| pDF29 | *steD::sgfp::pyrG* in *Sma*I site of pUC19 | This Study |
| pDF44 | *steC* genomic locus in *Pme*I site of pSK379 | This Study |
| pDF45 | *mkkB* genomic locus in *Pme*I site of pSK379 | This Study |
| pDF46 | *mpkB* genomic locus in *Pme*I site of pSK379 | This Study |
| pDF47 | *steD* genomic locus in *Pme*I site of pSK379 | This Study |
| pDF49 | *hamE* genomic locus in *Swa*I site of pOSB113 | This Study |
| pDF55 | *steC::sgfp::pyrG* in *Sma*I site of pUC19 | This Study |
