# Supplementary material for: The Pheromone Module SteC-MkkB-MpkB-SteD-HamE Regulates Development, Stress Responses and Secondary Metabolism in Aspergillus fumigatus
Source: Front Microbiol. 2020 May 7;11:811. doi: 10.3389/fmicb.2020.00811 (PMC7223695; doi:10.3389/fmicb.2020.00811)
Supplement: Supplementary file 4 [file Table_3.DOCX]

**Supplementary Table S3:** Oligonucleotides created or used in this study

| **Designation** | **Sequence in 5' > 3' direction** | **Size (basepairs)** |
| --- | --- | --- |
| DF13 (pDF4 5' UTR FWD, with pUC19 tail) | TTC GAG CTC GGT ACC CGA AAT GGC AGA AGT CTT CCA CAG | 39 |
| DF14 (pDF4 5' UTR REV, with *ptrA* tail) | GAT CCC GTA ATC AAT TTC TTG GGA TCT TGC GGT TGA G | 37 |
| DF15 (pDF4 3' UTR FWD, with *ptrA* tail) | AAA CAA AGA TGC AAG ACA CAG TCA CTT CTT TGA CTT GTC | 39 |
| DF16 (pDF4/5/6 3' UTR REV, with pUC19 tail) | ACT CTA GAG GAT CCC CCA TTG GCA CGG CGT ACC TG | 35 |
| DF17 (pDF4 5' FWD **nest oligo**) | GAA ATG GCA GAA GTC TTC CAC AG | 23 |
| DF18 (pDF4/5/6 3' REV **nest oligo**) | GCT GTG AAG CTG TAC TCG AC | 20 |
| DF19 (pDF5/6 *hamE* ORF FWD with pUC19 tail) | TTC GAG CTC GGT ACC CCT CAA AGA ACG CTC CGA CAG | 36 |
| DF20 (pDF5/6 5' FWD **nest oligo**) | GTG TTC TCC AGC TTC ACC TG | 20 |
| DF21 (pDF5/6 5' UTR REV, with tail for linker) | CAC CGC TAC CAC CTC CCA TCC GGC CGT CCG ACA TTT G | 37 |
| DF22 (pDF4 3' UTR FWD, with *hph* tail) | GAG GGC AAA GGA ATA GCA CAG TCA CTT CTT TGA CTT GTC | 39 |
| DF107 (pDF22 5' UTR REV, with *pyrG* tail) | GAG CAT TGT TTG AGG CAA CAT AAT ACC GGC GTT TTC CC | 38 |
| DF108 (pDF22 5' UTR FWD, with pUC19 tail) | TTC GAG CTC GGT ACC CGA GAG CGA TAG TTC CAA GTG G | 37 |
| DF109 (pDF22/26 3' UTR REV, with pUC19 tail) | ACT CTA GAG GAT CCC CCC TCT TGT TAT GCG GAG TGA G | 37 |
| DF110 (pDF22/26 3' UTR FWD, with *pyrG* tail) | GCC TCC TCT CAG ACA GGA TTT GAT ACT TTA TTG ATT GCA T | 40 |
| DF111 (pDF22 5' FWD **nest oligo**) | CAG CCA TCG AGT CGT GAT C | 19 |
| DF112 (pDF22/26 3' REV **nest oligo**) | GCA CGT GAC CAT CTG AGA C | 19 |
| DF113 (pDF23 5' UTR FWD, with pUC19 tail) | TTC GAG CTC GGT ACC CCC TCA TGA ACT TCG CCG TCG | 36 |
| DF114 (pDF23/27 3' UTR REV, with pUC19 tail) | ACT CTA GAG GAT CCC CCG TGA TAG CGA CCT TGA CGG | 36 |
| DF115 (pDF23 5' UTR REV, with *pyrG* tail) | GAG CAT TGT TTG AGG CGG CTG GGG CGA ACG GAG T | 34 |
| DF116 (pDF23/27 3' UTR FWD, with *pyrG* tail) | GCC TCC TCT CAG ACA GCA CCA TAC GTC GGA GCT ACT C | 37 |
| DF117 (pDF23 5' FWD **nest oligo**) | GAG TAC GGA GTA TGC TGC TG | 20 |
| DF118 (pDF23/27 3' REV **nest oligo**) | GAG TTG GCT TGG CAG GAA C | 19 |
| DF119 (pDF24 5' UTR FWD, with pUC19 tail) | TTC GAG CTC GGT ACC CCG ATC GAC GAA GCA GAA CAT TC | 38 |
| DF120 (pDF24/28 3' UTR REV, with pUC19 tail) | ACT CTA GAG GAT CCC CCC GAT CAA CTT ACT CTC CGA G | 37 |
| DF121 (pDF24 5' UTR REV, with *pyrG* tail) | GAG CAT TGT TTG AGG CGG TTG CTA ACT TTC AGC ATC | 36 |
| DF122 (pDF24/28 3' UTR FWD, with *pyrG* tail) | GCC TCC TCT CAG ACA GAG ATT CAG TGG CGT CTC TTG C | 37 |
| DF123 (pDF24 5' FWD **nest oligo**) | CGT GTG CCT GTC TTA CCT TAG | 21 |
| DF124 (pDF24/28 3' REV **nest oligo**) | GAG CTC AGC AAT CGA GCA ATC | 21 |
| DF125 (pDF25 5' UTR FWD, with pUC19 tail) | TTC GAG CTC GGT ACC CCA GAG CGC AGA GAT GTT GAG | 36 |
| DF126 (pDF25/29 3' UTR REV, with pUC19 tail) | ACT CTA GAG GAT CCC CCA GTA CCT GAA TAC ACT CGA GC | 38 |
| DF127 (pDF25 5' UTR REV, with *pyrG* tail) | GAG CAT TGT TTG AGG CGT CTA TGG AAG GGG GCT AG | 35 |
| DF128 (pDF25/29 3' UTR FWD, with *pyrG* tail) | GCC TCC TCT CAG ACA GTG CCA ACG TGC GCC TAG AC | 35 |
| DF129 (pDF25 5' FWD **nest oligo**) | CGA TGG TGA CGG AGC ATT GAG | 21 |
| DF130 (pDF25/29 3' REV **nest oligo**) | CCT TGG TCT CTG GGC TTG TC | 20 |
| DF134 (pDF27 5' UTR FWD, with pUC19 tail) | TTC GAG CTC GGT ACC CAT GGC CGA CCA GTT CAA AGC | 36 |
| DF135 (pDF27 5' UTR REV, with tail for linker) | CAC CGC TAC CAC CTC CGA CAG CTC CCG TCA TAT CG | 35 |
| DF136 (pDF27 5' FWD **nest oligo**) | CCA TTG GAA ACA CCG ACA GC | 20 |
| DF137 (pDF28 5' UTR FWD, with pUC19 tail) | TTC GAG CTC GGT ACC CCC AGG CGA CTC TCA CAA TTC | 36 |
| DF138 (pDF28 5' UTR REV, with tail for linker) | CAC CGC TAC CAC CTC CCC GCA TGA TTT CTT CGT AGA TC | 38 |
| DF139 (pDF28 5' FWD **nest oligo**) | CAC CAA CTC CTG TTC TGA GG | 20 |
| DF140 (pDF29 5' UTR FWD, with pUC19 tail) | TTC GAG CTC GGT ACC CGG ATG ACC TCG ACT GCG ATC | 36 |
| DF141 (pDF29 5' UTR REV, with tail for linker) | CAC CGC TAC CAC CTC CCA AAA CGC CGC CAG GTA GG | 35 |
| DF142 (pDF29 5' FWD **nest oligo**) | CGA CGT CCA CTC CAT TCA TC | 20 |
| DF259 (pDF44 5' *steC* ORF FWD, with pSK379 tail) | GCA GAC ATC ACC GTT TAT GTC ATT GAA GCC CAG TGC ATC | 39 |
| DF260 (pDF44 3' *steC* ORF REV, with pSK379 tail) | GAT AGA CAT GGC GTT TCT ACC GGA TAT TAA AAC GCC TTT | 39 |
| DF261 (pDF45 5' *mkkB* ORF FWD, with pSK379 tail) | GCA GAC ATC ACC GTT TAT GGC CGA CCA GTT CAA AGC | 36 |
| DF262 (pDF45 3' *mkkB* ORF REV, with pSK379 tail) | GAT AGA CAT GGC GTT TTT AGA CAG CTC CCG TCA TAT CG | 38 |
| DF263 (pDF46 5' *mpkB* ORF FWD, with pSK379 tail) | GCA GAC ATC ACC GTT TAT GGT GCA GCA ACC TCC TC | 35 |
| DF264 (pDF46 3' *mpkB* ORF REV, with pSK379 tail) | GAT AGA CAT GGC GTT TCT ACC GCA TGA TTT CTT CGT A | 37 |
| DF265 (pDF47 5' *steD* ORF FWD, with pSK379 tail) | GCA GAC ATC ACC GTT TAT GTC TCT GCA TAC ATC CTA CC | 38 |
| DF266 (pDF47 3' *steD* ORF REV, with pSK379 tail) | GAT AGA CAT GGC GTT TTC ACA AAA CGC CGC CAG GTA G | 37 |
| DF273 (pDF49 *hamE* 5' UTR FWD, with pOSB113 tail) | AGC TCG GTA CCC ATT TGT CTT CCA CAG GGC TGT TG | 35 |
| DF274 (pDF49 *hamE* 3' UTR REV, with pOSB113 tail) | TTG AGG CGA ATT ATT TGA AGC TGT ACT CGA CGA TTG AG | 38 |
| DF276 (pDF50 5’*steC* ORF FWD, with pCH008 tail) | GCC TGA GTG GCC GTT TAT GTC ATT GAA GCC CAG TGC | 36 |
| DF277 (pDF50 3’*steC* ORF REV, with tail for linker) | CTT GCT CAC CAT GTT TCC ACC GCT ACC ACC TCC CCG GAT ATT AAA ACG CCT TT | 53 |
| DF293 (pDF55 5' UTR FWD, with pUC19 tail) | TTC GAG CTC GGT ACC CGC GAA GGA TCA AGT AGG TCC | 36 |
| DF296 (pDF55 5’ UTR REV, with tail for linker) | CAC CGC TAC CAC CTC CCG CCA GCG GTG CAG ACA AAA AT | 38 |
| DF298 (pDF55 3’ UTR FWD, with tail for *pyrG*) | GCC TCC TCT CAG ACA GGA TGC ACC TGA ATT TCA GAA TT | 38 |
| DF299 (pDF55 3’ UTR REV, with tail for pUC19) | ACT CTA GAG GAT CCC CCC TTG AGT CAA GCT CTC TAA G | 37 |
| DF300 (pDF55 5’ FWD **nest oligo**) | CCA GTT CAA ACT GCG TTC GC | 20 |
| DF301 (pDF55 3’ REV **nest oligo**) | GCA GTT CAA ATG TGC GCA ACC | 22 |
